# Fresh Crab Plays an Important Role as a Nutrient Reservoir for the Rapid Propagation of *Vibrio vulnificus*

**DOI:** 10.3389/fmicb.2021.645860

**Published:** 2021-03-09

**Authors:** Suyeon Kim, Han Young Chung, Joon-Gi Kwon, Sang Ho Choi, Ju-Hoon Lee

**Affiliations:** ^1^National Research Laboratory of Molecular Microbiology and Toxicology, Department of Food and Animal Biotechnology, Department of Agricultural Biotechnology, Center for Food and Bioconvergence, Seoul National University, Seoul, South Korea; ^2^Food Microbiome Laboratory, Department of Food and Animal Biotechnology, Department of Agricultural Biotechnology, Center for Food and Bioconvergence, Seoul National University, Seoul, South Korea

**Keywords:** *Vibrio vulnificus*, fresh crab, food-borne pathogen, virulence factor, genomics, transcriptomics

## Abstract

*Vibrio vulnificus* is a well-known opportunistic pathogen causing food-borne illnesses by ingestion of contaminated seafood. A new strain of *V. vulnificus* FORC_016 was isolated from a patient’s blood sample in South Korea. The genome consists of two circular DNA chromosomes: chromosome I (3,234,424 bp with a G + C contents of 46.60% containing 2,889 ORFs, 106 tRNA genes, and 31 rRNA genes) and chromosome II (1,837,945 bp with a GC content of 47.00% containing 1,572 ORFs, 13 tRNA genes, and 3 rRNA genes). In addition, chromosome I has a super integron (SI) containing 209 ORFs, which is probably associated with various additional functions including antibiotic resistance and pathogenicity. Pan-genome analysis with other *V. vulnificus* genomes revealed that core genome regions contain most of the important virulence factors. However, accessory genome regions are located in the SI region and contain unique genes regarding cell wall biosynthesis and generation of host cell protecting capsule, suggesting possible resistance ability against environmental stresses. Comparative RNA-Seq analysis of samples between contact and no contact to the crab conditions showed that expressions of amino acid/peptide and carbohydrate transport and utilization genes were down-regulated, but expressions of cell division and growth-related genes were up-regulated, suggesting that the crab may be a nutrition reservoir for rapid propagation of *V. vulnificus*. Therefore, consumption of the contaminated fresh crab would provide a large number of *V. vulnificus* to humans, which may be more dangerous. Consequently, biocontrol of *V. vulnificus* may be critical to ensure the safety in seafood consumption.

## Introduction

*Vibrio vulnificus* is an opportunistic human pathogen that causes fever, nausea, septic shock, and its average mortality rate is over 50% (Jones and Oliver, 2009). It is found in coastal and estuarine environments worldwide, and various seafood including oysters, clams, crabs, and flounder are frequently contaminated with *V. vulnificus* (Horseman and Surani, 2011; Raszl et al., 2016; Heng et al., 2017). Human infections by this bacterium are mainly due to two contamination sources: ingestion of raw or undercooked seafoods (primary septicemia) and the exposure of wounds to seawater or seafood (wound infection) (Baker-Austin and Oliver, 2018). In the United States, the Food and Drug Administration (FDA) reported that in 459 cases of *V. vulnificus* infections, 51.6% of patients died between 1992 and 2007, indicating high fatality by the infection (Jones and Oliver, 2009). Therefore, many research groups have studied *V. vulnificus* to control its pathogenesis.

For the pathogenesis of *V. vulnificus*, most of the virulence factors are antibiotics resistance, adherence, host protection and antiphagocytosis, chemotaxis and mobility, exoenzyme, iron uptake, quorum sensing, and toxins. Among them, pili, flagella, capsular polysaccharides (CPS), cytolysin (*vvh*A), and repeats-in-toxins (RTX) are major virulence factors. For most bacterial infections, attachment and colonization of host surfaces are important steps in the early phase of infections. Pili are proteinaceous fibers that stick out from the cell surface of bacteria, which often mediate the initial attachment to the host surface. Likewise, *V. vulnificus* adherence has been demonstrated to correlate with increased piliation (Strom and Paranjpye, 2000). In addition, deletion of *pilA* and *pilD*, encoding pilin structural protein and pre-pilin peptidase, respectively, resulted in a loss of attachment to epithelial cells as well as a slight increase in LD_50_ (Paranjpye and Strom, 2005). Flagella-based motility has been proposed to be another virulence determinant in *V. vulnificus* (Lee et al., 2004). Loss of two flagella structural components, encoded by *flgC* and *flgE*, resulted in significant decreases in motility, cellular adhesion, and cytotoxicity when compared to a wild type (Kim and Rhee, 2003; Lee et al., 2004). The CPS protects *V. vulnificus* from phagocytosis by the host immune system. Inactivation of a CPS transport gene (*wza*) of *V. vulnificus* abolished capsule expression, resulting in decreased mortality (Wright et al., 2001). The extracellular cytolysin (VvhA) contributes to the virulence of *V. vulnificus* not only through the hemolytic activity, but also through other cytotoxic effects (Lohith et al., 2015). VvhA causes cell death by an increase of vascular permeability and hypotension via pore formation in the cellular membrane (Song et al., 2016). This cytolysin was demonstrated to cause severe tissue necrosis, fluid accumulation, intestinal irregularities, partial paralysis, and lethality. However, inactivation of VvhA did not affect the mortality of *V. vulnificus* in a mouse model, suggesting that it is not solely responsible for lethality and tissue damage (Yuan et al., 2020). RtxA is a multifunctional-autoprocessing repeats-in-toxin (MARTX) encoded by *rtxA*. The toxin is composed of repeated structural subunits, forming pores in host cellular membranes (Gulig et al., 2005; Satchell, 2015). Interestingly, RtxA of *V. vulnificus* is highly similar to that of *V. cholerae* with >80% sequence identity, suggesting a close evolutionary relationship (Lee et al., 2007; Kim et al., 2008). Mutation of *rtxA* showed lower cytotoxicity and higher LD_50_ than the wild type strain (Kim et al., 2008). In addition, RtxA is considered to trigger excessive production of reactive oxygen species (ROS) by the host cells, leading to necrotic cell death and apoptosis (Lee et al., 2007). RtxA also contributes to host cellular changes, including cytoskeleton rearrangement, bleb formation, and actin aggregation, which leads to cellular necrosis and enables *V. vulnificus* to invade the bloodstream by crossing the intestinal epithelium (Kim et al., 2008). Furthermore, RtxA and VvhA of *V. vulnificus* have been suggested to play an additional role in causing intestinal tissue damage and inflammation that promotes propagation of the infecting bacteria to the bloodstream and other organs (Jeong and Satchell, 2012).

An integron is a genetic element that contains mobile genes called cassettes (Rapa and Labbate, 2013). These genetic regions show multiple repeats and unusual guanine-cytosine content (GC) content and GC skew in the chromosome. Repeat sequence alignment showed a consensus sequence suggestive of a super integron (SI) cassette (Chen et al., 2003). The SI of the genus Vibrio contains an integrase (*intI*), a primary recombination site (*attI*), and multiple target-specific recombination sites (*attC*) (Fluit and Schmitz, 2004; Rapa and Labbate, 2013). The SI included several gene cassettes that encoded various adaptive functions, such as pathogenicity, resistance, and metabolism (Rowe-Maqnus et al., 1999). To further understand the pathogenesis mechanism of *V. vulnificus* and its transcription response to the food environment, additional omics studies of virulence factors may be required in genome and transcriptome levels.

While *V. vulnificus* is one of the major pathogens causing seafood outbreaks, its genome information and deduced functions are not fully understood yet. To date (April 2020), 22 complete genome sequences of *V. vulnificus* are available in the GenBank database. Among them, only three reports, with the complete genome sequences of strains MO6/24-O, FORC_017, and VV2014DJH, were published, showing general genome features with pathogenicity and various virulence factors predicted by bioinformatics (Chung et al., 2016; Pan et al., 2017). However, extended insights of transcriptional response between *V. vulnificus* and seafood environments are not understood yet. Because most infections of *V. vulnificus* are mediated by seafood consumption, it is also important to understand the changes of *V. vulnificus* gene expression profiles under exposure to given seafoods. This information would be useful to elucidate the survival and pathogenesis mechanisms in frequently contaminated seafood to control the pathogen for food safety.

In this study, *V. vulnificus* FORC_016, was isolated from an infected patient’s blood sample. To understand its survival and pathogenesis in the genome level, the genome was completely sequenced and various virulence factors were predicted. In addition, subsequent transcriptome analysis of FORC_016 was performed to elucidate its interaction and adaptation with a fresh crab sample for survival and pathogenesis. This study would be useful to extend the novel insight into its molecular pathogenic characteristics and correlative interaction between the pathogen and the crab environment in order to provide further information on the regulation of the pathogenicity of *V. vulnificus* in seafood.

## Results

### General Genome Properties of *V. vulnificus* FORC_016

**FIGURE 1:**
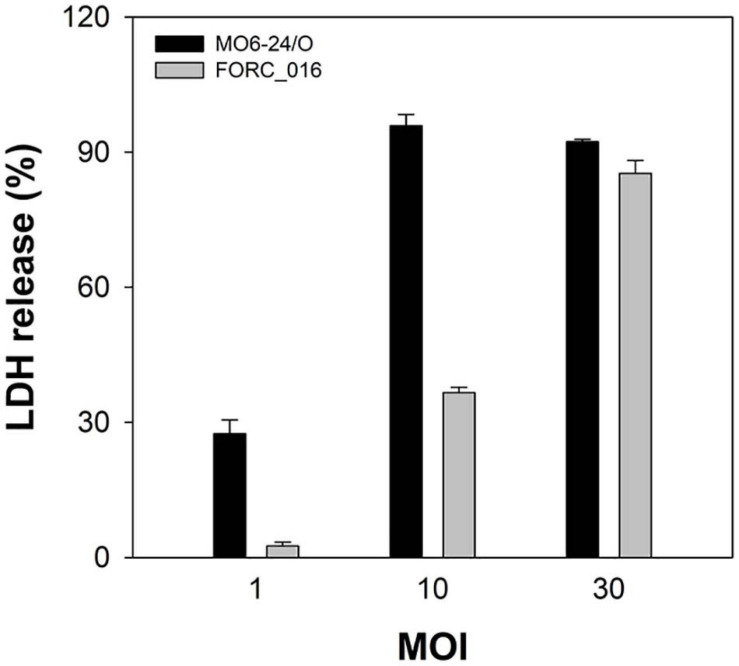
Cytotoxicity of *V. vulnificus* FORC_016. INT-407 cells were infected with *Vibrio* cells at various MOIs for 2 h. The cytotoxicity was determined by an LDH release assay. 100% LDH release is determined when 1% Triton X-100 was treated for complete lysis of the cells instead of the specific bacterium. Error bars represent the standard errors of the means (SEM). Cytotoxicity of *V. vulnificus* FORC_016 and *V. vulnificus* MO6-24/O (positive control).

**FIGURE 2:**
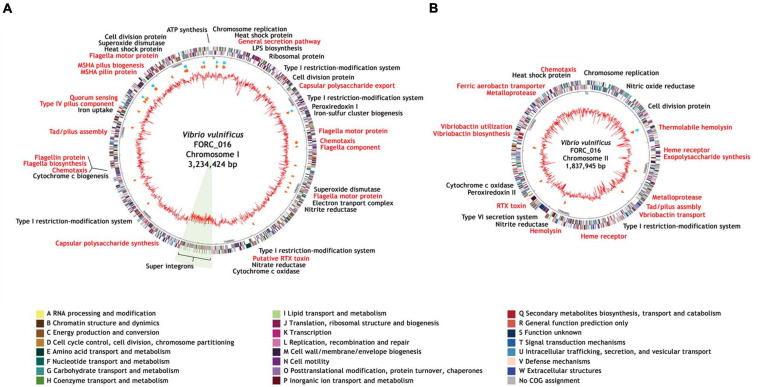
Genome maps of *V. vulnificus* FORC_016. **(A)** Genome map of the chromosome I. **(B)** Genome map of the chromosome II. The outer circle indicates the locations of all annotated ORFs, and the inner circle with the red peaks indicates GC content. Between these circles, sky blue arrows indicate the rRNA operons and orange arrows indicate the tRNAs. All annotated ORFs were colored differently according to the COG assignments. Genes with specialized functions labeled with different colors as follows: virulence-related genes in red and other functional genes in black.

*Vibrio vulnificus* FORC_016 was isolated from a contaminated patient’s blood sample by Chonbuk National University Hospital (Jeonju, South Korea). The cytotoxicity of FORC_016 was confirmed using the lactate dehydrogenase (LDH) release assays (Figure 1). To understand the virulence and toxicity at the genome level, the genomes were completely sequence and analyzed. Complete genome sequences of *V. vulfnicus* FORC_016 showed two circular double-stranded DNA chromosomes and no plasmid. Chromosome I consists of 3,234,424 bp with a G + C contents of 46.60% containing 2,889 ORFs, 106 tRNA genes, and 31 rRNA genes. Among them, 2,298 ORFs (79.54%) were assigned to be functional and 591 ORFs were hypothetical proteins. In addition, chromosome II consists of 1,837,945 bp with a GC content of 47.00% containing 1,572 ORFs, 13 tRNA genes, and 3 rRNA genes. Among them, 1,223 ORFs (77.80%) were assigned to be functional and 349 ORFs were hypothetical proteins. Furthermore, 2,711 ORFs on chromosome I and 1,453 ORFs on chromosome II were assigned into Clusters of Orthologous Groups (COGs) of proteins functional categories using the webMGA program (Wu et al., 2011). The general genome properties of *V. vulnificus* FORC_016 were summarized in Table 1, and the circular genome maps for FORC_016 were generated based on the genome information (Figure 2).

**TABLE 1:** Genome features of completely sequenced *V. vulnificus* strains and average nucleotide identity (ANI) values to FORC_016.

Strains	Total genome size (Mb)	Average G + C content (%)	No. of plasmids	No. of proteins	No. of tRNAs	No. of rRNAs	ANI value to FORC_016 (%)	Accession no.
								
FORC_016	5.07237	46.74	0	4,460	119	34	–	CP011775, CP011776
VV2014DJH	5.07456	46.84	0	4,513	120	37	98.125	CP019320, CP019321
FORC_017	5.22923	46.61	1	4,662	115	34	98.100	CP012739, CP012740
MO6-24/O	5.00777	46.95	0	4,453	111	28	98.090	CP002469, CP002470
FORC_077	5.01826	46.88	0	4,372	118	34	98.080	CP027030, CP027031
YJ016	5.26009	46.67	1	4,615	112	28	98.080	BA000037, BA000038
CMCP6	5.1267	46.72	0	4,516	112	28	98.075	AE016795, AE016796
FORC_036	6.06796	45.34	1	5,642	203	34	98.075	CP015512, CP015513
Env1	4.95405	46.74	0	4,363	118	34	98.065	CP017635, CP017636
FORC_053	6.01901	45.39	1	5,648	187	34	98.050	CP019290, CP019291
93U204	5.12735	46.70	1	4,452	107	24	98.045	CP009261, CP009262
FDAARGOS_119	4.9788	46.84	0	4,459	118	34	98.005	CP014048, CP014049
FDAARGOS_663	4.97482	46.71	0	4,354	118	34	95.055	CP044068, CP044069
ATCC_27562	5.00716	46.71	0	4,323	114	34	95.045	CP012881, CP012882
CECT_4999	5.16313	46.53	1	4,516	116	28	95.015	CP014636, CP014637
FORC_054	5.12077	46.68	1	4,577	118	34	94.975	CP019121, CP019122

Interestingly, *V. vulfnicus* FORC_016 has an unusually large DNA fragment with a low GC content (41.41%; FORC16_1435-1643) on chromosome I, which is a super integron region, suggesting that it may have been derived from a different origin (Figure 2A). Previously, the unusual DNA fragment has been identified as a super integron (SI) (FORC16_1435-FORC16_1643) containing 209 genes, which is probably associated with various additional functions including antibiotic resistance, and pathogenicity (Stokes and Hall, 1989; Rowe-Maqnus et al., 1999; Fluit and Schmitz, 2004; Naka et al., 2011). Nevertheless, their additional roles of SI in the genome of FORC_016 are not clearly understood, because most of the ORFs (62.57%) are hypothetical proteins.

FORC_016 has two chromosomes and each chromosome has its own virulence factors (Supplementary Table 2). Chromosome I has virulence factors regarding adherence (mannose-sensitive hemagglutinin (MSHA)-related genes and type IV pilus), chemotaxis and mobility (flagella biosynthesis and chemotaxis proteins), quorum sensing (autoinducer protein), and secretion system (general secretion pathway proteins), associated with dominant survival of a host strain under given environments. In addition, chromosome II has *Vibrio-*specific toxins (vibriolysin and cytolysin), antibiotic resistance (β-lactamase and related efflux pump), and iron uptake (TonB-dependent heme receptor and vibriobactin genes for ferric iron uptake), associated with the additional functions to compete with other bacteria. Therefore, the combination of these two chromosomes may cover all required basic and additional functions for dominant growth and survival of the host strain.

### Comparative Genome Analysis

**FIGURE 3:**
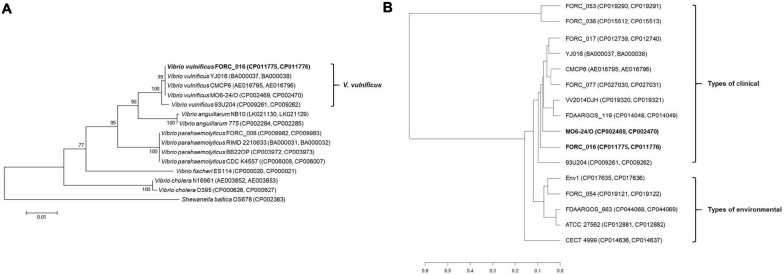
The 16S rRNA-based phylogenic tree analysis and ANI analysis of *Vibrio* species. **(A)** The 16S rRNA-based phylogenic tree of *V. vulnificus* FORC_016, and other 13 *Vibrio* species were constructed using the neighbor-joining method with 1,000 bootstrap replicates via MEGA6 software. *Shewanella baltica* OS678 was used as the outgroup. **(B)** ANI analysis of *Vibrio* species. The evolutionary relatedness between *V. vulnificus* species were measured using the average nucleotide identity values. The average nucleotide identity values were calculated using JSpecies by comparing whole genome sequences of *V. vulnificus*, which were fragmented into 1,020 bp, based on BLAST algorism. The trees were constructed using R programming.

16S rRNA-based phylogenetic tree analysis of *Vibrio* strains revealed that *V. vulnificus* FORC_016 was closely related to other *V. vulnificus* strains (Figure 3A). To further understand the evolutionary relationship among *V. vulnificus* strains, average nucleotide identity (ANI) analysis was conducted. The ANI analysis showed that FORC_016 belongs to the clinical type including other clinical *V. vulnificus* such as MO6-24/O, a clinical isolate from a patient with septicemia, suggesting that FORC_016 may be a potential pathogen causing foodborne outbreak (Figure 3B; Park et al., 2011). Interestingly, the ANI tree showed three groups of *V. vulnificus*. The first group contains only environmental genotype strains and the second group contains only clinical genotype strains. However, FORC_036 and FORC_053 are located in the outgroup of the tree (Figure 3B). Further genome analysis verified that they belong to the clinical genotype and their location in the ANI tree may be due to possession of their unique mega-plasmids. Therefore, *V. vulnificus* has two genotypes, environmental and clinical genotypes, and FORC_016 belongs to the clinical genotype in the ANI tree.

**FIGURE 4:**
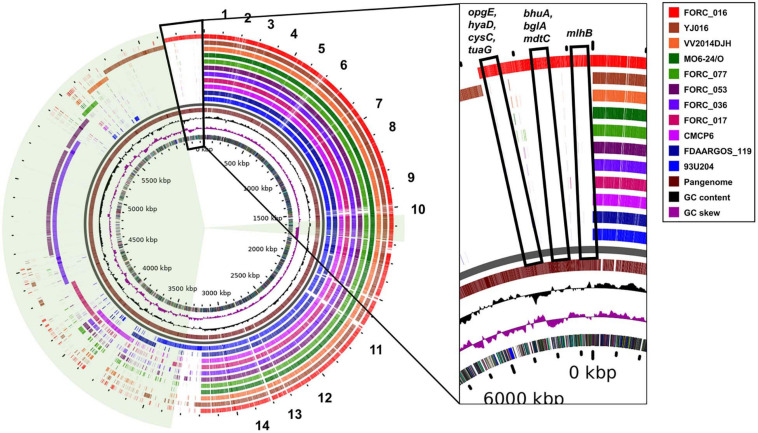
Circular view of pangenome of clinical isolated *V. vulnificus* genomes against the complete genome of reference strain MO6-24/O. 1, genes related with general secretion pathway; 2, genes related with polysaccharide export lipoprotein; 3, genes related with thermolabile hemolysin precursor and β-lactamase-related protein; 4, genes related with capsular polysaccharide synthesis enzyme; 5, genes related with chemotaxis protein; 6, genes related with ferric vibriobactin transport system and flagella biosynthesis; 7, genes related with flagella motor protein; 8, genes related with RTX toxin; 9, genes related with ferric vibriobactin transport system; 10, genes related with vibriolysin; 11, genes related with chemotaxis protein and flagellar biosynthesis; 12, genes related with type IV pilus component, autoinducer-2 production protein LuxS; 13, genes related with MSHA biogenesis protein; and 14, genes related with flagellar motor protein.

Complete genome sequences of 11 *V. vulnificus* strains in the clinical type were compared using pan-genome analysis to elucidate their differences in the genome level (Figure 4). These genomes share about 3.2 Mb-size chromosomes as a core genome. Most of the important virulence factors are located and well conserved in the core genome region of all *V. vulnificus* strains, suggesting that they may be key virulence factors for the host survival, biofilm formation, and toxin production (Figure 4). These key virulence factors include host survival genes (antibiotic resistance, adherence, chemotaxis, and iron uptake), biofilm formation genes (capsular polysaccharide synthesis), and toxin production genes (cytolysin, RTX toxin, vibriolysin, and hemolysin).

In addition, each strain has its own DNA region in the accessory genome region. Some of these unique genes in the region are homologous to 172 super integron genes of FORC_016 and scattered in the accessory genome region (Figure 4). However, 37 genes in the super integron region of FORC_016 are shared with other *V. vulnificus* genomes and are located in the core genome region, suggesting that they may play an important role in *V. vulnificus* (Figure 4). Nevertheless, these shared super integron genes encode hypothetical proteins, indicating that their exact functions are still unknown. Interestingly, FORC_036 and FORC_053 shared an approximately 700-kb large DNA region in the accessory genome region (4.6 Mb to 5.3 Mb in the map), which belongs to a mega-plasmid. Interestingly, FORC_016 has eight unique genes (*opgE, hyaD, cysC, tuaG, bhuA, bglA, mdtC*, and *mlhB*) in the pan-genome map (Figure 4). Among them, four unique genes (*opgE, hyaD, cysC*, and *tuaG*) may be associated with cell membrane biosynthesis (Tei et al., 1990; Bontemps-Gallo et al., 2013; Pinske and Sawers, 2014; Mirouze et al., 2018). In particular, *hyaD* gene encoding hyaluronan synthase was detected only in FORC_016 and may be responsible for the construction of a host cell protection capsule, suggesting possible resistance ability against environmental stress (Weigel, 2015).

### Pathogenesis and Virulence Factors of *V. vulnificus* FORC_016

*Vibrio vulnificus* is an opportunistic foodborne pathogen that can cause primary septicemia with a high mortality rate. Previous reports demonstrated that *V. vulniificus* has some important virulence factors, such as capsular polysaccharide synthesis enzyme (CPS; *cpsABCDFHIJ*), cytolysin (*vvhA*), RTX toxin (*rtxABCD*), and vibriolysin (*vvp*) (Lee et al., 2008; Song et al., 2016; Elgaml and Miyoshi, 2017; Pettis and Mukerji, 2020). The genome analysis revealed that FORC_016 has these virulence genes, suggesting it is a virulent pathogen that can cause a foodborne outbreak (Supplementary Table 2). Furthermore, the genome analysis of FORC_016 using the virulence factor database (VFDB) and the antibiotics resistance database (ARDB) detected and identified various potential virulence factors and were summarized in Supplementary Table 2 (Liu and Pop, 2009; Liu et al., 2019). They were categorized into several groups such as antibiotic resistance (β-lactamase), adherence (pilus biosynthesis), host protection and antiphagocytosis (capsular polysaccharide synthesis), chemotaxis and mobility (flagella biosynthesis and chemotaxis proteins), exoenzyme (vibriolysin), iron uptake (vibriobactin biosynthesis, transport, and utilization), quorum sensing, and toxins (cytolysin, RTX toxin, and hemolysin).

The genome of FORC_016 has antibiotic resistance-associated genes encoding a cyclic AMP receptor protein (FORC16_2625), β-lactamase (FORC16_3220), and a resistance-nodulation-division (RND) efflux system (FORC16_3925) (Supplementary Table 2). Cyclic AMP receptor protein is associated with the regulation of the efflux pump for fluoroquinolone-resistance activity (Kary et al., 2017) and β-lactamase is an enzyme for the inactivation of β-lactam antibiotics. Furthermore, the RND efflux system is a multidrug resistance efflux pump (Nikaido and Takatsuka, 2009). Therefore, FORC_016 may be resistant to broad-spectrum antibiotics. Capsular polysaccharide synthesis (CPS) plays an important role in bacterial capsule synthesis for host protection, biofilm formation, and resistance to opsonization by complement and subsequent phagocytosis (Lee et al., 2013; Pettis and Mukerji, 2020). The CPS gene cluster in the FORC_016 genome consists of two parts: CPS transport (FORC16_0182-0185) and capsular polysaccharide synthesis (CPS cluster I, FOR16_0186-0197; and CPS cluster II, FORC16_3287-3294) (Supplementary Table 2). Interestingly, while CPS cluster II is shared with other *V. vulnificus* strains, CPS cluster I may be strain-specific (Supplementary Figure 1A). Comparative genome analysis of CPS cluster I in FORC_016 and MO6-24/O showed no homology between the clusters (FORC16_0186-0197 vs. VVMO6_02777-02764), and thus supports this hypothesis.

Pathogenesis and survival of *V. vulnificus* in the host are highly associated with iron concentrations in infected individuals (Kim et al., 2007). In general, the *Vibrio* strain secretes vibriobactin as siderophore to bind to iron specifically and vibriobactin-mediated iron uptake and transport has been known to promote the host survival and pathogenesis (Tan et al., 2014). Genome analysis of FORC_016 revealed that it has vibriobactin biosynthesis genes (FORC16_4123, 4125, 4127-4131, and 4136-4137) and a strain-specific vibriobactin transport gene (*vctA;* FORC16_3496), suggesting the iron uptake ability of FORC_016 (Payne et al., 2015; Supplementary Figure 1B and Supplementary Table 2). In addition, FORC_016 has a strain-specific non-transferrin-bound iron transport gene (*hupA;* FORC16_3705) (Oh et al., 2009) (Supplementary Figure 1C). Therefore, FORC_016 has complete iron uptake and unique transport systems for survival and pathogenesis by dominant iron acquisition under given environments.

Bacterial quorum-sensing (QS) signaling is widely used to communicate with each other and to regulate the virulence genes under given environmental conditions (Shao et al., 2011). FORC_016 has a QS-associated gene (*luxS*) encoding autoinducer (FORC16_2386), suggesting its QS signaling activity for toxin or biofilm formation (Zhu et al., 2002). Vibriolysin from *V. vulnificus* is a major toxin that elicits the secondary wound damage for septicemia (Miyoshi, 2013). FORC_016 has a gene encoding of vibriolysin (FORC16_4281) and the metalloprotease with a hemorrhagic effect has been known to play an indirect role in the activation of other toxin proteins (Miyoshi et al., 2006). In addition, FORC_016 has other toxins such as cytolysin (*vvhA*), RTX toxin (*rtxABCD*), and hemolysin (*tlh*), suggesting its pathogenesis in human infection (Lo et al., 2011; Song et al., 2016). Therefore, it is necessary to elucidate the expression patterns of these virulence factors for further understanding of *V. vulnificus* FORC_016 adaptation and subsequent pathogenesis under given environmental conditions.

### Identification of Differentially Expressed Genes Under Exposure to Crab

**FIGURE 5:**
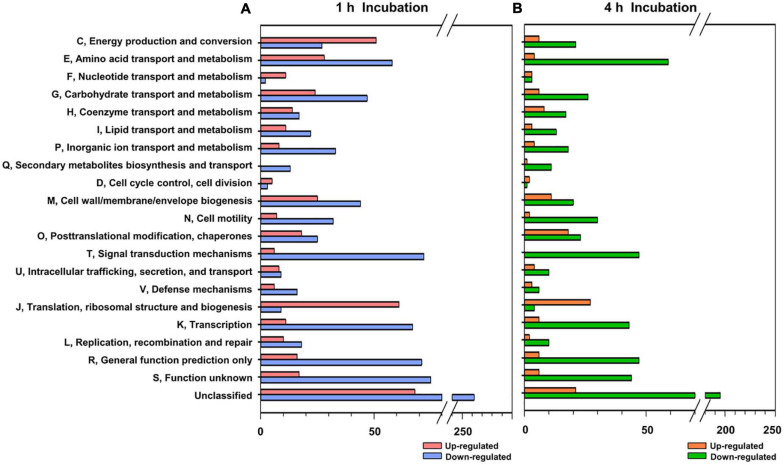
Functional categorization of differentially expressed genes. The differentially expressed genes (*P*-value < 0.05, two-fold threshold) were functionally categorized based on the COG database for the *V. vulnificus* FORC_016 genome, which was retrieved from GenBank (accession numbers CP011775 and CP011776). Genes up and down regulated by crab exposure for 1 h **(A)** and for 4 h **(B)**, were represented.

To further understand the interaction between FORC_016 and specific seafood, wild crab was selected and comparative RNA-Seq analysis via their interaction was performed. After reverse transcription of total mRNA and their cDNA sequencing using Illumina HiSeq 2500, about 56.7 million read sequences were obtained per sample and 88.9% of them were successfully mapped to a single location of a *V. vulnificus* FORC_016 reference genome. To elucidate the difference of RNA expression profiles between two conditions of FORC_016 with or without crab, their RNA expression profiles were compared after 1 or 4 h incubation (Supplementary Table 3). The comparative RNA-Seq analysis for 1,397 genes (405 up-regulated and 992 down-regulated) in 1 h incubation and for 791 genes (143 genes up-regulated and 648 gene down-regulated) in 4 h incubation was identified to be differentially expressed under given conditions. A subsequent COG database analysis grouped these genes into specific functional COG categories and then compared (Figure 5). Interestingly, amino acid transport and the metabolism category (E) revealed that most of the genes were down-regulated in both 1 and 4 h incubations (Figure 5). In addition, carbohydrate transport and the metabolism category (G) showed that most of the genes were down-regulated in both incubation conditions, which is probably associated with the conversion and down-regulation of gene expressions in energy production and the conversion category (C) from 1 h to 4 h incubations (Figure 5).

**FIGURE 6:**
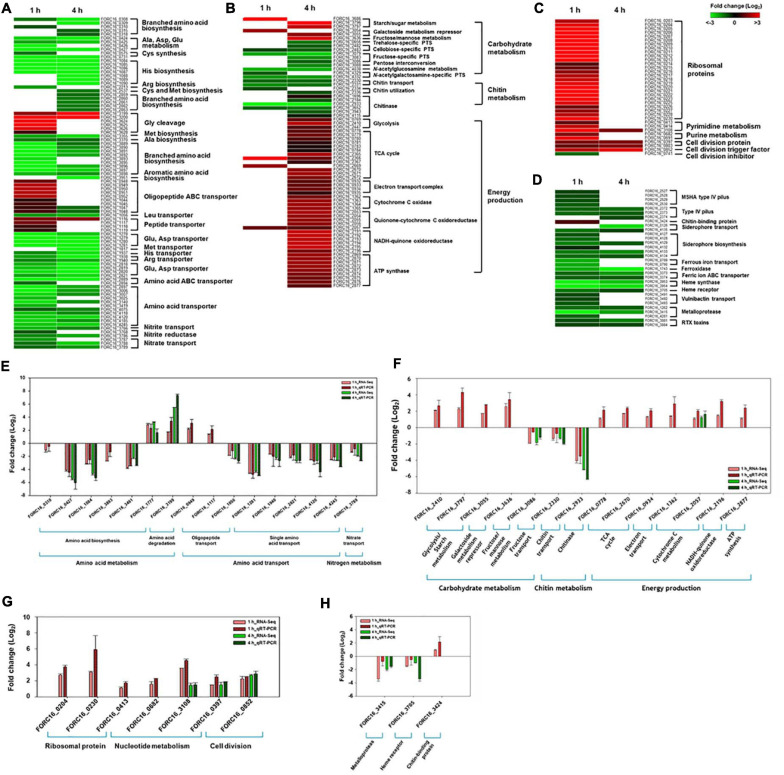
Heat maps of genes under exposure to crab. The expression ratio of genes related with amino acid metabolism between cells incubated with VFMG and VFMG containing crab for 1 and 4 h were calculated from RNA-seq results and demonstrated as heat maps (*P* < 0.05) **(A–D)**. **(A)** Genes related with amino acid metabolism. **(B)** Genes related with carbon metabolism and energy production. **(C)** Genes related with cell growth. **(D)** Genes related with virulence genes. The green-to-red color scale represented fold-change (log_2_). The results from RNA-seq were verified using qRT-PCR **(E–H)**.

Further comparative analysis of differentially expressed genes in category E showed that most of the genes associated with amino acid biosynthesis and amino acid transport were down-regulated during interaction between FORC_016 and the crab sample (Figure 6A). However, most of the genes associated with oligopeptide and peptide transporters as well as their degradation were up-regulated during this interaction, suggesting that the host strain may acquire peptides from the crab sample via their transporters, and then degrade them to obtain the amino acids required for protein biosynthesis (Figure 6A). Therefore, the host strain may not need to synthesize the amino acids via their transporters. Furthermore, nitrate/nitrite reductase-associated genes (FORC16_3768 and FORC16_3786) for nitrogen assimilation from nitrate (NO^3–^) and nitrite (NO^2–^) to ammonia as well as their transport genes were down-regulated. Ammonia is an essential element for amino acid biosynthesis (Mohiuddin and Khattar, 2020). The down-regulation of associated genes for nitrate/nitrite transport and ammonia production via nitrogen assimilation supports this hypothesis (Richardson et al., 2001; Reitzer, 2003; Mikami et al., 2017; Figure 6A). To verify the gene expression patterns for amino acid biosynthesis and transport, quantitative real-time PCR (qRT-PCR) analyses were performed. The qRT-PCR analysis showed the same patterns of comparative RNA-Seq results, thus substantiating this hypothesis (Figure 6E).

Previous carbohydrate composition analysis of blood tissue in green crab revealed that monosaccharides and glycogen were predominant sugars in crab tissue (Johnston and Davies, 1972). In this environment, specific gene expressions were up-regulated, regarding starch/sugar metabolism (FORC16_3686, FORC16_3796, and FORC16_3797) and fructose/mannose metabolism (FORC16_3636) (Figure 6B). Starch/sugar metabolism genes are related to degradation of sugar polymers and fructose/mannose metabolism genes are related to their phosphorylation for subsequent glycolysis after uptake of those sugars from the crab (Han et al., 2016). However, gene expression of other sugar-related genes was down-regulated, regarding the phosphotransferase system (PTS) for cellobiose (FORC16_2482-2483), trehalose (FORC16_0656), and N-acetylglucosamine (FORC16_4038), which is probably due to the abundance of glucose and glycogen in the crab environment (Johnston and Davies, 1972). However, galactoside transport and metabolism repressor genes (FORC16_3054-3055) were up-regulated, regarding the repression of galactoside transport and degradation, which is probably due to the direct uptake of galactose from the crab. In addition, FORC_016 does not utilize chitin, although the crab has lots of chitin and is supported by the down-regulation of chitin transport (FORC16_2330-2331) and utilization (FORC16_2334 and FORC16_2336) genes (Figure 6B). After the uptake of simple sugars and sugar polymers from the crab, they were utilized by glycolysis (FORC16_0769, FORC16_2410, and FORC16_2447) and the tricarboxylic acid (TCA) cycle (FORC16_0778-0784, FORC16_2365-2367, and FORC16_2669-2672) for production of reduced nicotinamide adenine dinucleotide (NADH) (Figure 6B). To produce energy as a form of ATP from NADH, the electron transport chain, consisting of NADH-quinone oxidoreductase (FORC16_2191-2196), quinone-cytochrome C oxidoreductase (FORC16_2053-2057), and cytochrome C oxidase (FORC16_1362-1365), produces the proton gradient by their proton pumping (Kracke et al., 2015). Therefore, all genes associated with the electron transport chain were up-regulated in the RNA level for activation of the electron transport chain (Figure 6B). In addition, the ATPsynthase gene (FORC16_2869-2877) was up-regulated because the proton motive force by proton gradient was used for ATP production via ATP synthase (Figure 6B). To verify these RNA expression profiles, qRT-PCR of these genes were performed. Interestingly, RNA expression patterns of RNA-Seq and qRT-PCR were exactly matched, indicating that these RNA expression profiles after contacting with the crab are correct (Figure 6F). Therefore, when *V. vulnificus* contaminated the fresh crab, it uptakes and utilizes most of the carbohydrates from the crab environment.

**FIGURE 7:**
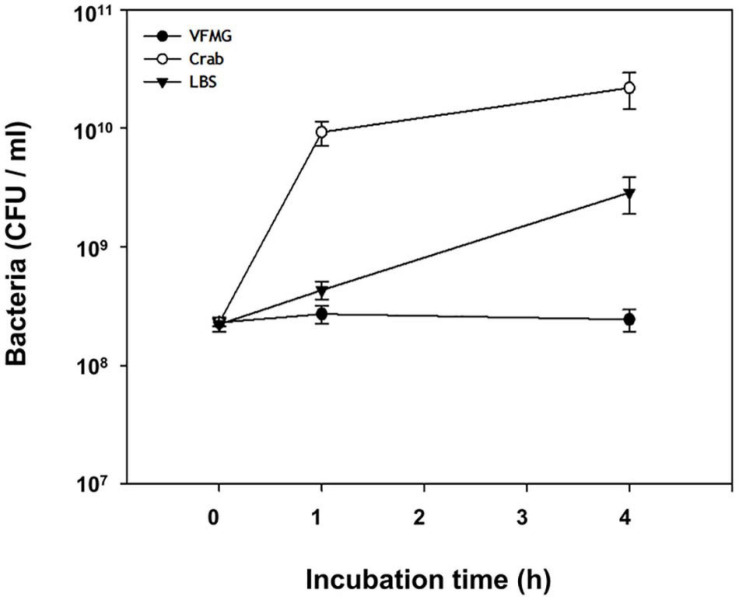
Growth of *V. vulnificus* on crab. *V. vulnificus* grown to A_600_ of 0.8 was harvested and washed, and subsequently inoculated in VFMG, VFMG containing crab, or LBS. Cell growth was measured at 1 and 4 h by CFU counting. Open circle, cells grown in VFMG; closed circle, cells grown in VFMG containing crab; and closed triangle, cells grown in LBS.

Bacterial cell growth may be associated with nucleotide biosynthesis for DNA replication and RNA synthesis, ribosome formation for translation, and biosynthesis of cell division proteins (Samant et al., 2008; Wang and Levin, 2009). When FORC_016 contacted with fresh crab, these bacterial cell growth-associate genes were up-regulated. In particular, expression of ribosomal protein genes (FORC16_0203-0230) significantly increased in the crab sample when compared to a negative control sample (no contact with crab), suggesting the activation of DNA and RNA biosynthesis as well as ribosome formation for cell growth (Xu et al., 2016; Figure 6C). In addition, the gene expressions of cell division protein FtsI (FORC16_0397), cell division topological specificity factor MinE (FORC16_0803), and the cell division trigger factor (FORC16_0852) were up-regulated, but the gene expression of the cell division inhibitor (FORC16_0741) were down-regulated, thus supporting this hypothesis (Wissel and Weiss, 2004; Jia et al., 2014). Therefore, the results of RNA-Seq analysis regarding cell growth suggest that contact with the fresh crab may activate the cell growth of FORC_016. To verify this hypothesis, FORC_016 was tested with two media (VFMG minimal medium and LBS-enriched medium) and a crab medium (VFMG medium with crab). Interestingly, viable cell counts of FORC_016 in the crab-added VFMG medium was much higher than those in only a VFMG minimal medium as well as the LBS-enriched medium (Figure 7). Comparing to the cells of FORC_016 in a VFMG minimal medium, those of FORC_016 in the VFMG medium with crab were grown up to a 1.8 log increment after 1 h incubation, and then up to a 2.0 log increment after 4 h incubation. In addition, the cell numbers of FORC_016 in the VFMG medium with crab were always higher than those in the LBS-enriched medium, substantiating that the fresh crab may provide much more nutrients (carbohydrates and oligopeptides) as well as enhancement of cell growth (Figure 7). Subsequent qRT-PCR analysis results for monitoring of these gene expressions between with and without crab conditions also support this hypothesis (Figure 6G).

Various virulence factors were detected in the genome annotation data (Supplementary Table 2) and their expression profiles were monitored using RNA-Seq analysis (Figure 6D). In particular, gene expressions in FORC_016 when contacted to a fresh crab, regarding chitin-binding protein, cell motility system, iron-associated uptake and transport systems, and RTX toxins, were compared with those expressions in the same strain with no contact to the crab. Interestingly, gene expression of only chitin-binding protein (FORC16_3424) was up-regulated, but gene expressions of type IV pilus-associated proteins (FORC16_2572-2530 and FORC16_2372-2374) were down-regulated, suggesting stable colonization of FORC_016 in the crab (Manjeet et al., 2013). In general, bacteria continuously compete with other bacteria in the same environment to dominantly uptake ferric/ferrous ions as an electron donor as well as acceptor in the biochemical redox reactions for energy production (Schröder et al., 2003). In addition, RNA-Seq analysis revealed that gene expressions of ferrous iron transport (FORC16_0789-0790), ferric ion ABC transport (FORC16_3373-3374), vulnibactin transport (FORC16_3491-3493), and siderophore biosynthesis and transport (FORC16_0126, FORC16_4127-4129, and FORC16_4132-4135) were highly down-regulated, suggesting that FORC_016 may uptake ferric/ferrous iron from the crab, but not from the environment by bacterial competition (Figure 6D). It was previously reported that the fresh green crab has plenty of iron (0.8 mg/100 g crab), thus supporting this hypothesis (Skonberg and Perkins, 2002). Interestingly, FORC_016 genome has genes of heme synthase and heme receptor (FORC16_3953-3954, and FORC16_3705), which may be useful for invasion and survival in the infected cells (Choby and Skaar, 2016). However, they were highly down-regulated, which is probably due to no heme in the crab (Figure 6D). In addition, RTX toxin of *V. vulnificus* is an important virulence factor for cell invasion and apoptosis by pore formation (Kim et al., 2008). However, gene expressions of RTX toxin (FORC16_3881 and FORC16_3884) were down-regulated in the crab environment, suggesting no toxin production of *V. vulnificus* (Figure 6D). Subsequent qRT-PCR analysis confirms these results (Figure 6H). Based on these results, FORC_016 may not produce virulence factors during its propagation in the crab.

## Discussion

*Vibrio vulnificus* is a well-known foodborne pathogen causing gastroenteritis and primary septicemia via consumption of contaminated seafoods (Heng et al., 2017). However, overall virulence factors and pathogenesis are not clearly understood even in the recent genomic era. To extend our knowledge about its genomic characteristics as well as virulence factors of *V. vulnificus*, the genomes of *V. vulnificus* FORC_016, isolated from the blood of a food-poisoning patient, which has a cytotoxicity as high as MO6-24/O, was completely sequenced and analyzed (Figure 1). The genomic analysis revealed that *V. vulnificus* FORC_016 has genes associated with CPS, cytolysin, and RTX toxins, which have been considered to be major virulence factors. Phylogenetic tree analysis using 16S rRNA gene sequences and further ANI analysis using genome sequences revealed that FORC_016 is closely related to clinical isolate strains, suggesting that the strain may be a potential pathogen. Furthermore, comparative genome analysis of *V. vulnificus* revealed that FORC_016 contains several strain-specific virulence factors contributing to the survival in the host.

It is well-known that *V. vulnificus* is a natural inhabitant in seawater and frequently contaminates seafood (Bisharat et al., 2020). The consumption of *V. vulnificus-*contaminated raw seafood causes food-borne illness to humans. However, the role of seafood for *V. vulnificus* is not fully understood yet, except for roles as a carrier as well as a reservoir of *V. vulnificus* from seawater to humans. Therefore, it is necessary to understand the role of seafood via the response analysis between *V. vulnificus* and seafood. In particular, gene expression patterns of *V. vulnificus-*specific virulence factors and toxins under seafood conditions should be investigated under a seafood environment. Therefore, to evaluate the roles of these virulence factors under seafood conditions, RNA-based transcriptome analysis may be required. In this study, the response via gene expression profiling of *V. vulnificus* FORC_016 to a specific seafood environment was monitored at the RNA level. Thus, comparative transcriptome profiling analysis was performed to identify differentially expressed virulence genes between VFMG and fresh crab conditions.

The transcriptome analysis under the fresh crab environment showed the upregulation of genes associated with the uptake and cleavage of oligopeptides, the uptake of simple sugars, glycolysis, the TCA cycle, energy production, and cell growth, and the downregulation of the uptake of amino acids, PTS for oligosaccharides, the uptake and utilization of chitin, and virulence factors. Interestingly, FORC_016 only uptakes oligopeptides and degrades them to amino acids in the cells, but does not uptake amino acids directly from the fresh crab (Figure 6A). In addition, FORC_016 uptakes starch, sugar, fructose, galactose, and mannose, and degrades starch and sugar to monosaccharides. With these simple sugars, FORC_016 utilizes glycolysis, the TCA cycle, and oxidative phosphorylation for ATP production (Stettner and Segrè, 2013). However, FORC_016 does not uptake and utilize some oligosaccharides and polysaccharides such as cellobiose, trehalose, and N-acetylglucosamine via PTS (Figure 6B). Through these mechanisms for acquisitions of amino acids, simple sugars from fresh crab and then ATP production may contribute to the upregulation of specific genes associated with translation as well as cell growth (Hironaka et al., 2013; Figure 6C). Additional bacterial growth curve analysis showed that the growth of FORC_016 in the fresh crab was much better than in both conditions of minimal and enriched media, and thus substantiating this hypothesis (Figure 7). Surprisingly, in this condition, the genes associated with various virulence factors and toxins were completely down-regulated, suggesting that *V. vulnificus* FORC_016 does not produce any virulence factors as well as toxins in fresh crab (Figure 6D). All transcriptome patterns were confirmed by qRT-PCR (Figures 6E–H). Therefore, these transcriptome profiling analysis revealed that *V. vulnificus* FORC_016 uptakes and utilizes oilgopeptides and simple sugars for energy production and its cell growth from the fresh crab. Even though its cell growth increases by the nutrient-rich fresh crab environment, FORC_016 does not produce virulence factors and toxins, suggesting that *V. vulnificus* might not be a life-threatening pathogen for fresh crab, and the role of fresh crab in foodborne illness is a reservoir of *V. vulnificus*. Previously, transcriptome analysis of *V. vulnificus* exposed to human serum relative to seawater was performed, which showed that *V. vulnificus* genes associated with virulence factors and toxins were upregulated under human serum conditions (Williams et al., 2014). This result suggests that *V. vulnificus* infection to humans via transmission with contaminated fresh crab could increase pathogenesis and toxin production in human conditions, even though genes regarding these virulence factors and toxins were completely down-regulated in the fresh crab. Consequently, transmission and pathogenesis strategies of *V. vulnificus* FORC_016 may really work for cell growth by nutrient uptake and energy production in fresh crab conditions and for increasing of pathogenesis after human infection using the contaminated crab as a carrier as well as a transmitter.

## Materials and Methods

### Strain Isolation, Identification, and Growth Conditions

*Vibrio vulnificus* FORC_016 was previously isolated from a patient’s blood sample in Jeonbuk National University Hospital (Jeonju, South Korea). It was identified using 16S rRNA gene sequencing with a 27F/1492R universal primer set (Acinas et al., 2004). A clinical isolate, *V. vulnificus* MO6-24/O was obtained from the culture collection of the Department of Food Science and Biotechnology, Seoul National University (South Korea). They were aerobically grown in modified Luria-Bertani medium supplemented with 2% (w/v) NaCl (LBS) for 12 h at 30°C. Agar medium was prepared with supplementation of 1.5% Bacto Agar (Difco, MI) (Choi et al., 2020).

### DNA and RNA Extraction

Genomic DNA was extracted and purified using a DNeasy Blood & Tissue Kit (Qiagen, Valencia, CA), according to the manufacturer’s protocol. Concentration of the purified genomic DNA was determined using a NanoVue spectrophotometer (GE Healthcare, Little Chalfont, United Kingdom). For RNA extraction, the bacterial cells grown to log phase (*A*_600_ of 0.8) were harvested and washed with sterilized phosphate-buffered saline (PBS). Then, PBS-resuspended cells (4 × 10^9^ CFU/250 ml) were subinoculated in either *Vibrio fischeri* minimal medium containing 32.6 mM sterilized glycerol (VFMG) with or without a tap water-prewashed fresh crab as test and control samples, respectively. These cultures were incubated for 1 or 4 h at 30°C. To remove the crab cells, the cultures were double-filtered using a syringe containing sterilized gauze and subsequent vacuum filter containing Whatman No.1 filter paper (Whatman, Maidstone, United Kingdom). After filtration, RNAprotect Bacteria Reagent (Qiagen, CA, United States) was added to the filtered solutions containing bacterial cells for RNA stability. Total RNAs were extracted and purified using a miRNeasy Mini Kit (Qiagen), according to the manufacturer’s procedure. The quality of total RNAs was determined using an Agilent 2100 Bioanalyzer and Agilent RNA 6000 Nano reagents (Agilent Technologies, Waldbronn, Germany).

### Cytotoxicity Test

To evaluate cytotoxicity of specific bacterium, the activity of cytoplasmic lactate dehydrogenase (LDH) released from damaged cells as an indicator of cell damage, was measured using a Cytotoxicity Detection Kit (Roche, Germany) with the manufacturer’s protocol. The INT-407 human intestinal epithelial cells (ATCC CCL-6) were incubated and tested with *V. vulnificus* FORC_016 and MO6-24/O at a various multiplicity of infection (MOI) for 2 h as previously described (Kim et al., 2014).

### Genome Sequencing and Bioinformatics Analysis

Bacterial complete genome sequencing was performed using the combination of an Illumina MiSeq platform (Illumina, United States) and a PacBio RS II platform (Pacific Biosciences, United States) according to their standard protocols. For construction of complete genome sequence, the qualified DNA reads were assembled using CLC Genomics Workbench ver. 7.0.4 (Qiagen Bioinformatics, United States) and PacBio SMRT Analysis ver. 2.0 software (Pacific Biosciences). The open reading frames (ORFs) and tRNA/rRNA of the assembled genome were predicted using a Rapid Annotation using Subsystem Technology (RAST) server (Aziz et al., 2008), and the predicted ORFs were manually curated with the ORF prediction result of the GeneMarkS program (Besemer et al., 2001). The ribosome binding sites (RBSs) of ORFs were predicted using RBSfinder (J. Craig Venter Institute, United States). For functional analysis and categorization of annotated ORFs, InterProScan5 with conserved protein domain databases (Zdobnov and Apweiler, 2001) and the COG-based WebMGA program (Wu et al., 2011) were used. Circular genome maps including all predicted ORFs with COG functional assignments, RNA operons, GC-content, and gene cluster information were generated using the GenVision program (DNASTAR, United States). Virulence factors and toxins were predicted using the Virulence Factor Database (VFDB; Liu et al., 2019), and antibiotic resistance genes were conducted using the Antibiotic Resistance Genes Database (ARDB; Liu and Pop, 2009). Phylogenetic tree analysis of *V. vulnificus* 16S rRNA genes were conducted using MEGA6 software with the neighbor-joining method and 1,000 bootstrap replicates (Tamura et al., 2013). Average Nucleotide Identity (ANI) analysis was performed using the JSpecies program^1^ based on BLAST algorism and the ANI tree was constructed using an R program (Richter and Rossello-Mora, 2009). Pan-genome analysis and its visualization were conducted using the BLAST Atlas program in a GView server^2^.

### Transcriptome Analysis

For cDNA synthesis, the extracted total mRNA was purified using Ribo-Zero rRNA Removal Kits (Epicentre, United States) to remove rRNAs and converted to cDNA using an iScript cDNA Synthesis Kit (Bio-Rad, United States). The cDNA library was evaluated using Bioanalyzer 2100 with DNA 1000 reagents (Agilent Technologies, United States). The cDNA sequencing was performed using an Illumina HiSeq 2500 sequencer. The cDNA sequence reads were mapped to complete genome sequences of *V. vulnificus* FORC_016, and the relative transcript abundance was measured with the concept of reads per kilobase of transcript per million mapped sequence reads (RPKM) (Mortazavi et al., 2008) using CLC Genomics Workbench 7.5.1 (Qiagen Bioinformatics). The fold changes of RPKM values and their significance were evaluated as previously described (Chung et al., 2018). The rectangular heat maps were generated by Gitools^3^ based on the fold changes of RPKM values of each gene.

### Quantitative Real-Time (qRT) PCR

For quantification of specific mRNA transcripts, quantitative real-time PCR (qRT-PCR) amplification was performed using the Chromo 4 real-time PCR detection system (Bio-Rad) with SYBR Green I and a pair of specific primers listed in Supplementary Table 1. Relative expression levels of the specific transcripts were calculated by using the 16S rRNA expression level as the internal reference for normalization (Kim et al., 2011).

### Growth Kinetics of FORC_016

*Vibrio vulnificus* FORC_016 grown to *A*_600_ of 0.8 was harvested and washed, and subsequently inoculated in VFMG, VFMG containing crab, or LBS. The growth of *V. vulnificus* was measured by counting the colony forming unit (CFU)/ml from serial dilutions by 1X PBS plated onto LBS agar. The incubation times were 0 h, 1 h, and 4 h. The plates were incubated in 30°C for 12 h and the colonies were counted. The experiments were run with biological duplicates and for each biological sample, technical triplicates were used.

### Nucleotide Sequence Accession Numbers

Complete genome sequences of *V. vulnificus* FORC_016 were deposited in the GenBank database of the National Center for Biotechnology Information (NCBI)^4^ under accession numbers CP011775 and CP011776. Total mRNA sequences for transcriptome analysis in *V. vulnificus* FORC_016 were deposited in the NCBI Sequence Read Archive (SRA)^5^ under accession number PRJNA532503.

## Data Availability Statement

The datasets presented in this study can be found in online repositories. The names of the repository/repositories and accession number(s) can be found in the article/Supplementary Material.

## Author Contributions

SC and J-HL designed the research. SK, HC, and J-GK performed the research. SK, HC, J-GK, and J-HL analyzed the data. SK, HC, SC, and J-HL wrote the manuscript. All authors contributed to the article and approved the submitted version.

## Conflict of Interest

The authors declare that the research was conducted in the absence of any commercial or financial relationships that could be construed as a potential conflict of interest.

## Footnotes

1 http://imedea.uib-csic.es/jspecies/

2 https://server.gview.ca/

3 http://www.gitools.org

4 http://www.ncbi.nlm.nih.gov/genbank

5 http://www.ncbi.nlm.nih.gov/sra
