# Supplementary material for: Fresh Crab Plays an Important Role as a Nutrient Reservoir for the Rapid Propagation of Vibrio vulnificus
Source: Front Microbiol. 2021 Mar 9;12:645860. doi: 10.3389/fmicb.2021.645860 (PMC7985530; doi:10.3389/fmicb.2021.645860)
Supplement: Supplementary file 1 [file Presentation_1.pptx]

## Slide 1
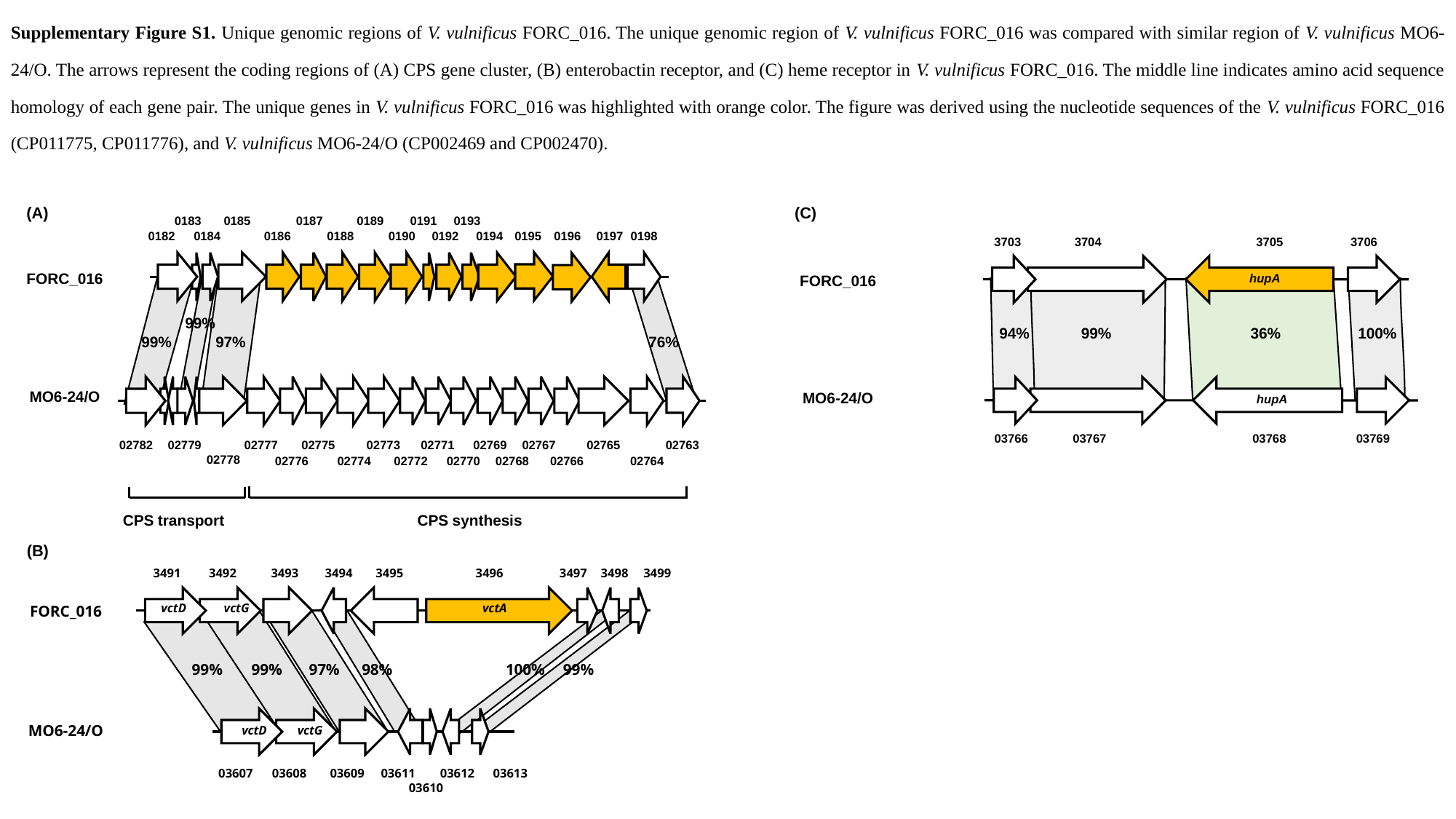

Supplementary Figure S1. Unique genomic regions of V. vulnificus FORC_016. The unique genomic region of V. vulnificus FORC_016 was compared with similar region of V. vulnificus MO6-24/O. The arrows represent the coding regions of (A) CPS gene cluster, (B) enterobactin receptor, and (C) heme receptor in V. vulnificus FORC_016. The middle line indicates amino acid sequence homology of each gene pair. The unique genes in V. vulnificus FORC_016 was highlighted with orange color. The figure was derived using the nucleotide sequences of the V. vulnificus FORC_016 (CP011775, CP011776), and V. vulnificus MO6-24/O (CP002469 and CP002470).
(A)
0183
0185
0187
0189
0191
0193
0182
0184
0186
0188
0190
0192
0194
0195
0196
0197
0198
‘
‘
‘
‘
‘
FORC_016
MO6-24/O
99%
99%
97%
76%
‘
‘
‘
‘
‘
‘
‘
‘
‘
‘
‘
‘
‘
‘
‘
‘
‘
‘
‘
‘
‘
02782
02779
02763
02777
02775
02773
02771
02769
02767
02765
02778
02774
02772
02770
02768
02766
02764
02776
CPS transport
CPS synthesis
(C)
3703
3704
3705
3706
‘
‘
hupA
94%
99%
36%
100%
‘
‘
hupA
03766
03767
03768
03769
FORC_016
MO6-24/O
(B)
3499
3498
3491
3492
3493
3494
3495
3496
3497
‘
‘
‘
vctA
vctD
vctG
99%
99%
97%
98%
100%
99%
‘
‘
‘
vctD
vctG
03613
03612
03607
03608
03609
03611
03610
FORC_016
MO6-24/O
