# Supplementary material for: Fresh Crab Plays an Important Role as a Nutrient Reservoir for the Rapid Propagation of Vibrio vulnificus
Source: Front Microbiol. 2021 Mar 9;12:645860. doi: 10.3389/fmicb.2021.645860 (PMC7985530; doi:10.3389/fmicb.2021.645860)

**Supplementary Data**

**Fresh Crab Plays an Important Role as a Nutrient Reservoir for the Rapid Propagation of *Vibrio vulnificus***

**Suyeon Kim^1†^, Han Young Chung^­1†^, Joon-Gi Kwon^2^, Sang Ho Choi^­1*^, and**

**Ju-Hoon Lee^2*^**

^1^ National Research Laboratory of Molecular Microbiology and Toxicology, Department of Food and Animal Biotechnology, Department of Agricultural Biotechnology, Center for Food and Bioconvergence, Seoul National University, Seoul, South Korea

^2^ Food Microbiome Laboratory, Department of Food and Animal Biotechnology, Department of Agricultural Biotechnology, Center for Food and Bioconvergence, Seoul National University, Seoul, South Korea

**Running title: *Vibrio vulnificus* infection to crab**

*Correspondence:

Dr. Sang Ho Choi

choish@snu.ac.kr

Dr. Ju-Hoon Lee

juhlee@snu.ac.kr

**Supplementary Table S1.** General genome properties of *V. vulnificus* FORC_016

**Supplementary Table S2.** Detected virulence factors of *V. vulnificus* FORC_016 against the Antibiotic Resistance Database (ARDB) and the Virulence Factor Database (VFDB)

**Supplementary Table S3.** List of genes differentially expressed by the exposure of a swimming crab

**Supplementary Figure S1.** Unique genomic regions of *V. vulnificus* FORC_016. The unique genomic region of *V. vulnificus* FORC_016 was compared with a similar region of *V. vulnificus* MO6-24/O. The arrows represent the coding regions of (A) CPS gene cluster, (B) enterobactin receptor, and (C) heme receptor in *V. vulnificus* FORC_016. The middle line indicates amino acid sequence homology of each gene pair. The unique genes in *V. vulnificus* FORC_016 was highlighted with orange color. The figure was derived using the nucleotide sequences of *V. vulnificus* FORC_016 (CP011775, CP011776), and *V. vulnificus* MO6-24/O (CP002469 and CP002470).

**Supplementary Table S1.** General genome properties of *V. vulnificus* FORC_016

|  | **Sequence (5' → 3')** | |
| --- | --- | --- |
| **Locus-tag** | **Forward** | **Reverse** |
| **FORC16_0204** | AATCGGTCGTAAAGTGGGCA | ATTGCTGCATAGCCGTCAGT |
| **FORC16_0230** | ACAGTGTTGCTAACCGTCGT | TTGGAGCTTTGTCACCAGCA |
| **FORC16_0310** | TTCGGACGAGTGTGCAATGA | CATGAGAGCGGCTGAAAGGA |
| **FORC16_0397** | GCCAGTTAGCGATCCGAGAA | TCATCGGGTGCCACATTCAA |
| **FORC16_0413** | ACCTCGATGACGGGGTATCA | GAGATCGCGAATCACAAGGC |
| **FORC16_0427** | GGGAACTCGGTCTTGCAGAA | AGCACCTAATAGCGTTGCGA |
| **FORC16_0682** | GGTTATGCTCGCCAAGATCG | AATCGTCAGAACGCGGTCAA |
| **FORC16_0741** | TCTCGAAACACCTCACGCTC | CTTGGGCGTCCAGAGAACAT |
| **FORC16_0778** | TTGTTGATTCGTGCAACGGC | ACAGAAGCGAGAGCAAGCAT |
| **FORC16_0815** | GGTGATTGGCGCAACCAAAT | TAAGCCAGCCACTTCTTCCG |
| **FORC16_0852** | CGTCGTTTCGATGGTTTCCG | ACCCGCTGGGTTGATCTTTT |
| **FORC16_0934** | GCATTACCCGGATTGTTGGC | CCAGAGCACTATGATCGCGT |
| **FORC16_0948** | AGCCAGAAAACTTCGTCGGT | CAACCGCGTTTTCGATTGGT |
| **FORC16_0949** | TTGCGCTGCATTGGGATCTA | GGTAGCTCAACCCTTTCGCT |
| **FORC16_1047** | CTGATTTGCGACGAACCCAC | ATATCTCCGCAACGACACCG |
| **FORC16_1056** | GTGGGGCTTTCTCCAACTCC | AACAAACACGAGCAACGAGG |
| **FORC16_1084** | CGGCGATGGCTTATGGTACA | GTACTTCTGATGGCCCAGCG |
| **FORC16_1117** | TCATCGGCCACGACTTAACC | GCTGCGGTATAGGGATGCTT |
| **FORC16_1281** | CGCTTTACCGCTCTGCAATC | AAAACCTTGGCCGTCGTAGT |
| **FORC16_1362** | GTTGGCAACTATTGGCACAGT | AGCCTCAACAGATTCCACGA |
| **FORC16_1777** | GTAGCCCAGAGCAAGTGTGT | GAAGTGCCATGCGTGTTGAG |
| **FORC16_1940** | GTGACGCATGAAATGGCGTT | AGCGCTCTGAATCAGGGTTA |
| **FORC16_2057** | CAGAAGTTGGTCAGCGCCTA | CGGAACAAGTCTGGCAGGAT |
| **FORC16_2196** | CTCTAGCGGGTCCTGTTGTG | GTAAGCGTGAGGACCTGTGG |
| **FORC16_2330** | GTATTTTCGCAGCATGGCGT | GGGAACCAACCAAGACCGAT |
| **FORC16_2366** | GCAGAAGCGGCTCCAGTA | TGATGGAATCGCCAACAGCC |
| **FORC16_2410** | GAGCTACGTGACGGTGACAA | GTGCCGTCTAGCTCGATCAT |
| **FORC16_2482** | GAAAGAGTTTGTGCTGGCGG | ATCCTGCGCATGAACCATGA |
| **FORC16_2670** | GCGCCTAGAAGCGATCAAAC | GTTCAAACCGAACTGCGGAC |
| **FORC16_2821** | TCAGCGTGTAGTCATTGCGA | ATAGCTCGCGGATCAGGTTG |
| **FORC16_2859** | TAAACTGCGTCATGCCAATGC | GTGTTGTCCGGCATGAGGT |
| **FORC16_2877** | AGTCCGGCGCGGTTATTTTT | CGCTCTCGCTCCTACAAACA |
| **FORC16_2933** | GGTGTGGGTCACGACAAGAT | GACCAGGGCGCATGAAGTTA |
| **FORC16_3007** | TGACGGTTTCTACTTCGAATAAACG | AAGTCGATCTCCCCCGCTT |
| **FORC16_3055** | CCTGATTGTCACCCAAGGCA | AGCACACGCAAATGATCACC |
| **FORC16_3086** | CTAGGCTCACTAACAACGGCG | AACAGGTGACTGCGACGAT |
| **FORC16_3108** | AGCCATCACCGTTTGCAGTA | GATCAACTCACCCAGTGCGA |
| **FORC16_3199** | AGATTGCCAGTGGCGAAGAA | CAGATAAAGCGCACAGACGC |
| **FORC16_3449** | AATAGCGACAAACCTGGGCA | GGTAAAGGTCTGCTTCCCCC |
| **FORC16_3636** | AGCCTAGCCAACAAAGGCAT | AGCATAAGCCGGCCACTAAG |
| **FORC16_3643** | GCAGTACTTGATGGCACGGA | TGCCGAGATCATCGAGGAGA |
| **FORC16_3766** | ATCATTCTGTCGTTGGCGGT | TTTCGCTAACGGTGCCAAAC |
| **FORC16_3789** | CACAAAGGCGACAACAGTGG | GGCACGCCAATGCTCTTAAA |
| **FORC16_3797** | TGAAGAGCTGGCTTACGCTT | GTTATGGAAGCGCAGCATGT |
| **FORC16_3872** | GGCTAGTGAGGAGCGTCTTG | GACCAATGCCAACCCCAAAC |
| **FORC16_3887** | CTTGCACGTTACGGCATACG | CATCCACTTCCAGCACTCGT |
| **FORC16_3891** | TGAGGTGCTCAAAGAGGCTG | GTCATAAAGGCGGTGGTGGA |
| **FORC16_3893** | GTTTACCGTTGCACCCGTTT | TCACTTTATCCGCGGCCAG |
| **FORC16_4076** | TTACCTCAAACGTCGCGGAA | TGGACGCTTTAAACGGCTCT |
| **FORC16_4120** | AGATCCTTGGGCTCCGTTTG | ATTCAAGGCTCTGGTCCACG |
| **FORC16_4245** | ACGCATTAAACGCTCAGGGA | ATAGGTTTCATCGGCCGCAA |
| **FORC16_4394** | GGGTGCTGTGGGTCAGTTTA | CTTCATCACCGCTTTCCAGC |
| **FORC16_4407** | AGTGATCAAGCCAGACGCAA | GTTGCGGTTCATGCCTTTCC |

**Supplementary Table S2.** Detected virulence factors of *V. vulnificus* FORC_016 against the Antibiotic Resistance Database (ARDB) and the Virulence Factor Database (VFDB)

| **Virulence factor** | **Annotation** | **Chromosome** | **Location** | **Function** |
| --- | --- | --- | --- | --- |
| **Antibiotic** |  |  |  |  |
| FORC16_2625 | Cyclic AMP receptor protein | Chromosome I | 2932416-2933048  (FORC16_2625) | Antibiotic efflux |
| *varG* | Beta-lactamase-related protein | Chromosome II | 386062-387192  (FORC16_3220) | Antibiotic inactivation |
| *adeF* | RND efflux system,  inner membrane transporter CmeB | Chromosome II | 1228638-1231790  (FORC16_3925) | Antibiotic efflux |
| **Adherence** |  |  |  |  |
| *mshH, mshI, mshJ, mshK, mshL, mshM, mshN, mshE, mshG, mshF* | MSHA biogenesis protein | Chromosome I | 2808331-2819759  (FORC16_2521-FORC16_2530) | Hemagglutinin activity |
| *mshB, mshA, mshC, mshD* | MSHA pilin protein | Chromosome I | 2806055-2808252  (FORC16_2516-FORC16_2520) | Hemagglutinin activity |
| *pilA, pilB, pilC, pilD* | Type IV pilus component | Chromosome I | 2643815-2648192  (FORC16_2372-FORC16_2375) | Adhesion, motility |
| **Antiphagocytosis** | | | | |
| *wza, wzb, wzc* | Polysaccharide export lipoprotein | Chromosome I | 205204-209818  (FORC16_0182-FORC16_0185) | Bacterial protection |
| *cpsA, cpsB, cpsC, cpsD, cpsF, cpsH, cpsI, cpsJ* | Capsular polysaccharide  synthesis enzyme | Chromosome II | 483745-493549  (FORC16_3287-FORC16_3294) | Bacterial protection |
| **Chemotaxis and motility** | | | | |
| *flaC, flaA, flgL, flgK, flgJ, flgI, flgH, flgG, flgF, flgE, flgD, flgC, flgB, flgA, flgM, flgN, flhB, fliR, fliQ, flip, fliO, fliN, fliM, fliL, fliK, fliJ, fliI, fliH, fliG, fliF, fliE, flrC, flrB, flrA, fliS, flaI, fliD, flaG, flaB, flaD, flaE, fliA, flhG, flhF, flhA* | Flagellar biosynthesis | Chromosome I | 788191-802388  (FORC16_0712-FORC16_0726) 784485-785725  (FORC16_0707-FORC16_0709) 2309235-2334475  (FORC16_2074-FORC16_2098) 2303801-2309074  (FORC16_2070-FORC16_2073) | Flagella component, biosynthesis |
| *cheR, cheV, cheW, cheB, cheA, cheZ, cheY* | Chemotaxis protein | Chromosome I | 785801-787568  (FORC16_0710-FORC16_0711) 2296755-2303756  (FORC16_2062-FORC16_2069) | Chemotaxis |
| *motA, motB, motX, motY* | Flagellar motor protein | Chromosome I | 703980-705710  (FORC16_0634-FORC16_0635) 1029858-1030739  (FORC16_0928) 2952903-2953547  (FORC16_2640) | Flagella component, Motility |
| **Exoenzyme** | | | | |
| *hap/vvp* | Vibriolysin | Chromosome II | 1633754-1635583  (FORC16_4281) | Metalloprotease,  hemorrhagic effect |
| **Iron uptake** |  |  |  |  |
| *hutA, hutR* | TonB-dependent heme receptor | Chromosome II | 451296-453431  (FORC16_3262) 959076-961241  (FORC16_3705) | Ferric ion (heme) uptake |
| *vctC, vctD, vctG, vctP* | Ferric vibriobactin  transport system | Chromosome II | 704225-706854  (FORC16_3491-FORC16_3493) 1469691-1470599  (FORC16_4135) | Ferric ion uptake |
| *vibA, vibB, vibC, vibD, vibE, vibF, vibH* | Vibriobactin biosynthesis | Chromosome II | 1451002-1451697  (FORC16_4123)  1454829-1459313  (FORC16_4125)  1461006-1464994  (FORC16_4127- FORC16_4129)  1465991-1466878  (FORC16_4131) 1473103-1474374  (FORC16_4137) | Ferric ion uptake |
| *viuA, viuB* | Vulnibactin  (Vibriobactin utilization) | Chromosome II | 1465112-1465927  (FORC16_4130)  1470699-1472762  (FORC16_4136) | Ferric ion uptake |
| **Quorum sensing** |  |  |  |  |
| *luxS* | Autoinducer-2 production protein LuxS | Chromosome I | 2656199-2656717  (FORC16_2386) | Autoinducer production |
| **Secretion system** |  |  |  |  |
| *epsC, gspD, epsE, epsF, epsG, epsH, epsI, epsJ, epsK, epsL, epsM, epsN* | General secretion pathway | Chromosome I | 117640-128959  (FORC16_0095-FORC16_0106) | Secretion |
| **Toxin** |  |  |  |  |
| *vvhA* | Cytolysin precursor | Chromosome II | 1077146-1078561  (FORC16_3801) | Cytotoxicity |
| *rtxA, rtxC, rtxB, rtxD* | RTX toxin | Chromosome II | 1168015-1188077  (FORC16_3881-FORC16_3885) | Toxin, Actin monomer  cross-linking activity |
| *tlh* | Thermolabile hemolysin precursor | Chromosome II | 367907-369160  (FORC16_3198) | Lipase activity |

**Supplementary Table S3.** List of genes differentially expressed by the exposure of a swimming crab

| **Locus tag^a^** | **Gene product** | **1h** | | | | | **4h** | | | | |
| --- | --- | --- | --- | --- | --- | --- | --- | --- | --- | --- | --- |
|  |  | **Fold change** | | | ***P v*alue** | | **Fold change** | | ***P v*alue** | |  |
| **Amino metabolism** | | | | | | | | | | | |
| FORC16_0308 | 3-isopropylmalate dehydratase small subunit | -1.466 | | 0.031 | | | -2.881 | | | 0.026 | |
| FORC16_0309 | 3-isopropylmalate dehydratase large subunit |  | |  | | | -2.947 | | | 0.044 | |
| FORC16_0310 | 3-isopropylmalate dehydrogenase | -1.009 | | 0.044 | | |  | | |  | |
| FORC16_0315 | Probable transcriptional activator for leuABCD operon |  | |  | | | -1.244 | | | 0.014 | |
| FORC16_0318 | Acetolactate synthase small subunit |  | |  | | | -1.209 | | | 0.049 | |
| FORC16_0424 | Glutamate synthase (NADPH) small chain | -2.578 | | 0.013 | | | -3.569 | | | 0.003 | |
| FORC16_0425 | Glutamate synthase (NADPH) large chain | -2.068 | | 0.01 | | | -2.475 | | | 0.026 | |
| FORC16_0426 | Glutamate synthase (NADPH) small chain | -3.762 | | 0.031 | | |  | | |  | |
| FORC16_0427 | Glutamate synthase (NADPH) large chain | -4.078 | | 0.029 | | | -5.427 | | | 0.017 | |
| FORC16_0575 | D-amino acid dehydrogenase small subunit | -1.423 | | 0.008 | | |  | | |  | |
| FORC16_0731 | Cysteine synthase | -3.784 | | 0.009 | | | -5.444 | | | 0.011 | |
| FORC16_1084 | Histidinol dehydrogenase | -3.117 | | 0.013 | | | -4.65 | | | 0.043 | |
| FORC16_1085 | Histidinol-phosphate aminotransferase | -3.54 | | 0.004 | | | -5.213 | | | 0.029 | |
| FORC16_1086 | Histidinol-phosphatase / Imidazoleglycerol-phosphate dehydratase | -3.778 | | 0.02 | | | -5.469 | | | 0.031 | |
| FORC16_1087 | Imidazole glycerol phosphate synthase amidotransferase subunit | -3.787 | | 0.039 | | | -5.579 | | | 0.025 | |
| FORC16_1088 | Phosphoribosylformimino-5-aminoimidazole carboxamide ribotide isomerase |  | |  | | | -5.638 | | | 0.023 | |
| FORC16_1089 | Imidazole glycerol phosphate synthase cyclase subunit |  | |  | | | -5.521 | | | 0.02 | |
| FORC16_1090 | Phosphoribosyl-AMP cyclohydrolase / Phosphoribosyl-ATP pyrophosphatase |  | |  | | | -5.374 | | | 0.016 | |
| FORC16_2213 | N-acetylglutamate synthase | -1.899 | | 0.006 | | |  | | |  | |
| FORC16_2537 | Aspartokinase |  | |  | | | -1.531 | | | 0.021 | |
| FORC16_2859 | Acetolactate synthase large subunit |  | |  | | | -1.33 | | | 0.007 | |
| FORC16_2860 | Acetolactate synthase small subunit |  | |  | | | -1.503 | | | 0.019 | |
| FORC16_2861 | Branched-chain amino acid aminotransferase |  | |  | | | -1.579 | | | 0.024 | |
| FORC16_2862 | Dihydroxy-acid dehydratase |  | |  | | | -1.503 | | | 0.017 | |
| FORC16_2863 | Threonine dehydratase biosynthetic |  | |  | | | -1.467 | | | 0.037 | |
| FORC16_3199 | 2-amino-3-ketobutyrate coenzyme A ligase | 1.733 | | 0.019 | | | 5.501 | | | 0.015 | |
| FORC16_3200 | L-threonine 3-dehydrogenase | 3.024 | | 0.021 | | | 5.768 | | | 0.014 | |
| FORC16_3355 | Peptide methionine sulfoxide reductase MsrA / Peptide methionine sulfoxide reductase MsrB | -2.032 | | 0.009 | | | -2.67 | | | 0.003 | |
| FORC16_3370 | 4-aminobutyrate aminotransferase | -1.51 | | 0.004 | | | -1.322 | | | 0.022 | |
| FORC16_3889 | Enoyl-CoA hydratase (valine, isoleucine degradation) | -3.667 | | 0.042 | | | -1.449 | | | 0.028 | |
| FORC16_3890 | Enoyl-CoA hydratase (valine, isoleucine degradation) | -4.048 | | 0.022 | | | -1.775 | | | 0.005 | |
| FORC16_3891 | Branched-chain acyl-CoA dehydrogenase | -3.746 | | 0.039 | | | -2.196 | | | 0.005 | |
| FORC16_3892 | Methylmalonate-semialdehyde dehydrogenase | -3.304 | | 0.019 | | | -2.087 | | | 0.022 | |
| FORC16_3893 | 3-ketoacyl-CoA thiolase (isoleucine degradation) | -2.692 | | 0.002 | | |  | | |  | |
| FORC16_3895 | Isovaleryl-CoA dehydrogenase | -3.854 | | 0.001 | | |  | | |  | |
| FORC16_3896 | Methylcrotonyl-CoA carboxylase carboxyl transferase subunit | -4.031 | | 0.013 | | | -2.271 | | | 0.003 | |
| FORC16_3897 | Methylglutaconyl-CoA hydratase | -3.143 | | 0.008 | | | -2.608 | | | 0.013 | |
| FORC16_3898 | Hydroxymethylglutaryl-CoA lyase | -2.277 | | 0.008 | | | -1.844 | | | 0.008 | |
| FORC16_3951 | 2-keto-3-deoxy-D-arabino-heptulosonate-7- phosphate synthase I alpha |  | |  | | | -3.026 | | | 0.002 | |
| **Amino acid transport** | | | | | | | | | | | |
| FORC16_0948 | Oligopeptide ABC transporter, periplasmic oligopeptide-binding protein OppA | 1.322 | | 0.007 | | |  | | |  | |
| FORC16_0949 | Oligopeptide transport system permease protein OppB | 2.186 | | 0.003 | | |  | | |  | |
| FORC16_0950 | Oligopeptide transport system permease protein oppC | 2.179 | | 0.003 | | |  | | |  | |
| FORC16_0951 | Oligopeptide transport ATP-binding protein OppD | 2.157 | | 0.004 | | |  | | |  | |
| FORC16_0952 | Oligopeptide transport ATP-binding protein OppF | 2.078 | | 0.003 | | |  | | |  | |
| FORC16_1047 | Oligopeptide transport ATP-binding protein OppD | 0.671 | | 0.026 | | | -1.336 | | | 0.043 | |
| FORC16_1048 | Oligopeptide transport ATP-binding protein OppF | 0.815 | | 0.03 | | | -1.351 | | | 0.027 | |
| FORC16_1056 | Leucine-responsive regulatory protein, regulator for leucine (or lrp) regulon and high-affinity branched-chain amino acid transport system | -1.839 | | 0.018 | | | -2.235 | | | 0.014 | |
| FORC16_1116 | Peptide transport system ATP-binding protein SapF | 1.476 | | 0.004 | | | 1.574 | | | 0.05 | |
| FORC16_1117 | Peptide transport system ATP-binding protein SapD | 1.376 | | 0.002 | | |  | | |  | |
| FORC16_1118 | Peptide transport system permease protein SapC | 0.734 | | 0.005 | | |  | | |  | |
| FORC16_1119 | Peptide transport system permease protein SapB | 0.605 | | 0.013 | | |  | | |  | |
| FORC16_1278 | ABC-type polar amino acid transport system, ATPase component | -2.99 | | 0.039 | | | -2.457 | | | 0.004 | |
| FORC16_1279 | Glutamate Aspartate transport system permease protein GltK | -3.351 | | 0.035 | | | -3.159 | | | 0.007 | |
| FORC16_1280 | Glutamate Aspartate transport system permease protein GltJ | -3.784 | | 0.016 | | | -3.68 | | | 0.001 | |
| FORC16_1281 | Glutamate Aspartate periplasmic binding protein precursor GltI | -4.584 | | 0.009 | | | -4.245 | | | 0.011 | |
| FORC16_1817 | Methionine ABC transporter ATP-binding protein |  | |  | | | -3.301 | | | 0.001 | |
| FORC16_1937 | Histidine ABC transporter, permease protein HisM | -1.779 | | 0.012 | | | -2.789 | | | 0.005 | |
| FORC16_1938 | Arginine/ornithine ABC transporter, permease protein AotQ | -1.996 | | 0.002 | | | -3.158 | | | 0.009 | |
| FORC16_1940 | Arginine/ornithine ABC transporter, ATP-binding protein AotP | -1.652 | | 0.00035081 | | | -2.169 | | | 0.027 | |
| FORC16_2818 | Dipeptide transport system permease protein DppC | -2.204 | | 0.001 | | | -3.344 | | | 0.003 | |
| FORC16_2819 | ABC transporter permease | -2.407 | | 0.009 | | | -3.603 | | | 0.002 | |
| FORC16_2820 | Dipeptide-binding ABC transporter, periplasmic substrate-binding component | -2.759 | | 0.017 | | | -3.789 | | | 0.023 | |
| FORC16_2821 | Dipeptide transport ATP-binding protein DppD | -2.093 | | 0.037 | | | -2.695 | | | 0.022 | |
| FORC16_2889 | Amino acid ABC transporter, periplasmic amino acid-binding portion | -3.463 | | 0.043 | | | -5.568 | | | 0.003 | |
| FORC16_2894 | Peptide ABC transporter, ATP-binding protein | -1.296 | | 0.007 | | |  | | |  | |
| FORC16_2897 | ABC-type amino acid transport/signal transduction system | -3.344 | | 0.037 | | | -2.132 | | | 0.011 | |
| FORC16_3006 | ABC-type amino acid transport/signal transduction system | -3.255 | | 0.012 | | | -2.031 | | | 0.002 | |
| FORC16_3007 | ABC-type amino acid transport/signal transduction system | -2.64 | | 0.004 | | | -1.78 | | | 0.009 | |
| FORC16_3025 | Oligopeptide transport system permease protein OppB | -2.311 | | 0.002 | | |  | | |  | |
| FORC16_3140 | ABC-type amino acid transport, signal transduction systems, periplasmic component/domain | -2.795 | | 0.005 | | | -1.872 | | | 0.015 | |
| FORC16_3419 | Serine transporter | -2.2 | | 0.028 | | |  | | |  | |
| FORC16_4075 | Amino acid transporter | -1.148 | | 0.018 | | | -1.646 | | | 0.013 | |
| FORC16_4118 | ABC-type amino acid transport/signal transduction systems | -2.359 | | 0.025 | | | -1.719 | | | 0.014 | |
| FORC16_4120 | Putative amino acid ABC transporter, periplasmic amino acid-binding protein | -2.487 | | 0.025 | | | -2.683 | | | 0.047 | |
| FORC16_4185 | ABC-type amino acid transport/signal transduction system | -2.558 | | 0.037 | | | -1.848 | | | 0.009 | |
| FORC16_4245 | ABC-type branched-chain amino acid transport system, periplasmic component | -2.458 | | 0.025 | | | -2.592 | | | 0.008 | |
| **Nitrogen metabolism** | | | | | | | | | | | |
| FORC16_3766 | Nitrite transporter from formate/nitrite family | -2.241 | | 0.004 | | | -1.645 | | | 0.006 | |
| FORC16_3768 | Nitrite reductase (NAD(P)H) large subunit | -1.089 | | 0.046 | | |  | | |  | |
| FORC16_3786 | Nitrite reductase (NAD(P)H) large subunit | -1.622 | | 0.035 | | | -2.079 | | | 0.003 | |
| FORC16_3787 | Nitrate ABC transporter, ATP-binding protein | -1.952 | | 0.003 | | |  | | |  | |
| FORC16_3788 | Nitrate ABC transporter, permease protein | -1.397 | | 0.034 | | | -2.522 | | | 0.013 | |
| FORC16_3789 | Nitrate ABC transporter, nitrate-binding protein | -1.291 | | 0.001 | | | -1.851 | | | 0.035 | |
| **Carbon metabolism** | | | | | | | | | | | |
| FORC16_0769 | Phosphoglucomutase | 1.166 | | 0.016 | | |  | | |  | |
| FORC16_2410 | Enolase | 2.122 | | 0.001 | | |  | | |  | |
| FORC16_2447 | Fructose-bisphosphate aldolase class II | 0.993 | | 0.017 | | |  | | |  | |
| FORC16_2482 | PTS system, cellobiose-specific IIA component | -1.128 | | 0.024 | | |  | | |  | |
| FORC16_2483 | PTS system, cellobiose-specific IIC component | -1.216 | | 0.006 | | | -0.842 | | | 0.045 | |
| FORC16_3686 | Glucan 1,6-alpha-glucosidase |  | |  | | | 4.006 | | | 0.024 | |
| FORC16_3796 | Glycosidase | 3.239 | | 0.00013838 | | |  | | |  | |
| FORC16_3797 | Glycosidase | 2.294 | | 0.016 | | |  | | |  | |
| FORC16_3054 | Galactose operon repressor, GalR-LacI family of transcriptional regulators |  | |  | | | 1.827 | | | 0.033 | |
| FORC16_3055 | Galactose/methyl galactoside ABC transport system, permease protein MglC | 1.728 | | 0.039 | | |  | | |  | |
| FORC16_3636 | Tagatose-6-phosphate kinase / 1-phosphofructokinase | 2.57 | | 0.047 | | |  | | |  | |
| FORC16_0656 | PTS system, trehalose-specific IIB component / PTS system, trehalose-specific IIC component | -1.469 | | 0.033 | | |  | | |  | |
| FORC16_2482 | PTS system, cellobiose-specific IIA component | -1.128 | | 0.024 | | |  | | |  | |
| FORC16_2483 | PTS system, cellobiose-specific IIC component | -1.216 | | 0.006 | | | -0.842 | | | 0.045 | |
| FORC16_3079 | PTS system, fructose-specific IIA component / PTS system, fructose-specific IIB component / PTS system, fructose-specific IIC component | -2.176 | | 0.002 | | | -2.208 | | | 0.001 | |
| FORC16_3083 | PTS system, fructose-specific IIA component / PTS system, fructose-specific IIB component / PTS system, fructose-specific IIC component |  |  | | | -1.139 | |  |  |  |  |
| FORC16_3086 | PTS system, fructose-specific IIBC component | -1.92 | | 0.005 | | | -1.84 | | | 0.005 | |
| FORC16_3088 | 4-deoxy-L-threo-5-hexosulose-uronate ketol-isomerase | -2.561 | | 0.009 | | | -2.034 | | | 0.017 | |
| FORC16_3653 | Glucose-1-phosphate adenylyltransferase | -2.602 | | 0.022 | | |  | | |  | |
| FORC16_4038 | N-acetylmannosamine-6-phosphate 2-epimerase | -1.744 | | 0.023 | | |  | | |  | |
| FORC16_4329 | N-acetylglucosamine-6-phosphate deacetylase | -1.255 | | 0.046 | | |  | | |  | |
| FORC16_4332 | PTS system, N-acetylgalactosamine-specific IIC component | -1.653 | | 0.046 | | |  | | |  | |
| **Chitine metabolism** | | | | | | | | | | | |
| FORC16_2330 | (GlcNAc)2 ABC transporter, permease component 1 | -1.312 | | 0.027 | | | -1.303 | | | 0.028 | |
| FORC16_2331 | (GlcNAc)2 ABC transporter, permease component 2 | -1.445 | | 0.047 | | | -1.739 | | | 0.042 | |
| FORC16_2334 | Glucosamine-link cellobiase |  | |  | | | -0.955 | | | 0.029 | |
| FORC16_2336 | Beta-N-acetylhexosaminidase, (GlcNAc)2 catabolism | -0.992 | | 0.012 | | |  | | |  | |
| FORC16_1696 | Chitinase | 0.489 | | 0.009 | | |  | | |  | |
| FORC16_2184 | Chitinase | -1.04 | | 0.017 | | |  | | |  | |
| FORC16_2933 | Chitinase | -4.054 | | 0.01 | | | -5.125 | | | 0 | |
| FORC16_3652 | Chitodextrinase precursor | -1.467 | | 0.011 | | | -1.226 | | | 0.018 | |
| FORC16_3943 | Chitinase | -1.128 | | 0.03 | | | -0.86 | | | 0.083 | |
| FORC16_4115 | Chitinase | -0.447 | | 0.035 | | | -0.765 | | | 0.082 | |
| **Energy production** | | | | | | | | | | | |
| FORC16_0778 | Succinate dehydrogenase hydrophobic membrane anchor protein | 1.098 | | 0.012 | | |  | | |  | |
| FORC16_0779 | Succinate dehydrogenase flavoprotein subunit | 1.389 | | 0.001 | | |  | | |  | |
| FORC16_2365 | Dihydrolipoamide dehydrogenase of pyruvate dehydrogenase complex | 2.03 | | 0.00031143 | | |  | | |  | |
| FORC16_2366 | Dihydrolipoamide acetyltransferase component of pyruvate dehydrogenase complex | 1.241 | | 0.004 | | | 2.963 | | | 0.039 | |
| FORC16_2669 | Succinate dehydrogenase flavoprotein subunit |  | |  | | | 1.742 | | | 0.046 | |
| FORC16_2670 | Succinate dehydrogenase iron-sulfur protein | 1.721 | | 0.001 | | |  | | |  | |
| FORC16_2671 | Fumarate reductase subunit C | 1.936 | | 0.00015635 | | |  | | |  | |
| FORC16_2672 | Fumarate reductase subunit D | 1.553 | | 1.7703E-05 | | |  | | |  | |
| FORC16_0932 | Electron transport complex protein RnfE | 1.112 | | 0.004 | | |  | | |  | |
| FORC16_0933 | Electron transport complex protein RnfG | 1.192 | | 0.002 | | |  | | |  | |
| FORC16_0934 | Electron transport complex protein RnfD | 1.299 | | 0.001 | | |  | | |  | |
| FORC16_0935 | Electron transport complex protein RnfC | 0.610 | | 0.002 | | |  | | |  | |
| FORC16_1362 | Cytochrome c oxidase subunit CcoN | 1.414 | | 0.008 | | |  | | |  | |
| FORC16_1363 | Cytochrome c oxidase subunit CcoO | 1.834 | | 0.013 | | |  | | |  | |
| FORC16_1364 | Cytochrome c oxidase subunit CcoQ | 1.892 | | 0.014 | | |  | | |  | |
| FORC16_1365 | Cytochrome c oxidase subunit CcoP | 1.827 | | 0.009 | | |  | | |  | |
| FORC16_2053 | Cytochrome c heme lyase subunit CcmH | 1.987 | | 0.001 | | |  | | |  | |
| FORC16_2054 | Cytochrome c heme lyase subunit CcmL | 1.774 | | 0.00030488 | | |  | | |  | |
| FORC16_2055 | Cytochrome c-type biogenesis protein CcmG/DsbE, thiol:disulfide oxidoreductase | 1.74 | | 0.001 | | |  | | |  | |
| FORC16_2056 | Cytochrome c heme lyase subunit CcmF | 1.653 | | 0.00040254 | | |  | | |  | |
| FORC16_2057 | Cytochrome c-type biogenesis protein CcmE, heme chaperone | 1.059 | | 0.026 | | | 1.244 | | | 0.022 | |
| FORC16_2191 | Na(+)-translocating NADH-quinone reductase subunit F | 2.347 | | 0.002 | | |  | | |  | |
| FORC16_2192 | Na(+)-translocating NADH-quinone reductase subunit E | 2.31 | | 0.001 | | |  | | |  | |
| FORC16_2193 | Na(+)-translocating NADH-quinone reductase subunit D | 2.193 | | 0.002 | | |  | | |  | |
| FORC16_2194 | Na(+)-translocating NADH-quinone reductase subunit C | 2.34 | | 0.003 | | |  | | |  | |
| FORC16_2195 | Na(+)-translocating NADH-quinone reductase subunit B | 2.364 | | 0.001 | | |  | | |  | |
| FORC16_2196 | Na(+)-translocating NADH-quinone reductase subunit A | 1.468 | | 0.003 | | |  | | |  | |
| FORC16_2869 | ATP synthase epsilon chain | 1.812 | | 0.005 | | |  | | |  | |
| FORC16_2870 | ATP synthase beta chain | 1.806 | | 0.005 | | |  | | |  | |
| FORC16_2871 | ATP synthase gamma chain | 1.859 | | 0.004 | | |  | | |  | |
| FORC16_2872 | ATP synthase alpha chain | 1.812 | | 0.005 | | |  | | |  | |
| FORC16_2873 | ATP synthase delta chain | 1.67 | | 0.009 | | |  | | |  | |
| FORC16_2874 | ATP synthase B chain | 1.489 | | 0.011 | | |  | | |  | |
| FORC16_2875 | ATP synthase F0 sector subunit c | 1.34 | | 0.022 | | |  | | |  | |
| FORC16_2876 | ATP synthase F0 sector subunit a | 1.438 | | 0.017 | | |  | | |  | |
| FORC16_2877 | ATP synthase protein I2 | 1.099 | | 0.038 | | |  | | |  | |
| **Ribosomal protein** | | | | | | | | | | | |
| FORC16_0203 | SSU ribosomal protein S10p (S20e) | 2.147 | | 1.028 E-04 | | |  | | |  | |
| FORC16_0204 | LSU ribosomal protein L3p (L3e) | 2.726 | | 4.496 E-04 | | |  | | |  | |
| FORC16_0205 | LSU ribosomal protein L4p (L1e) | 3.315 | | 1.305 E-04 | | |  | | |  | |
| FORC16_0206 | LSU ribosomal protein L23p (L23Ae) | 3.568 | | 2.265 E-04 | | |  | | |  | |
| FORC16_0207 | LSU ribosomal protein L2p (L8e) | 3.749 | | 1.901 E-04 | | |  | | |  | |
| FORC16_0208 | MULTISPECIES: 30S ribosomal protein S19 | 3.892 | | 2.583 E-04 | | |  | | |  | |
| FORC16_0209 | LSU ribosomal protein L22p (L17e) | 4.123 | | 8.171 E-05 | | |  | | |  | |
| FORC16_0210 | SSU ribosomal protein S3p (S3e) | 4.222 | | 2.250 E-05 | | |  | | |  | |
| FORC16_0211 | LSU ribosomal protein L16p (L10e) | 4.435 | | 1.930 E-04 | | |  | | |  | |
| FORC16_0212 | LSU ribosomal protein L29p (L35e) | 4.348 | | 2.585 E-04 | | |  | | |  | |
| FORC16_0213 | SSU ribosomal protein S17p (S11e) | 4.366 | | 7.545 E-05 | | |  | | |  | |
| FORC16_0214 | LSU ribosomal protein L14p (L23e) | 1.063 | | 1.850 E-04 | | |  | | |  | |
| FORC16_0215 | LSU ribosomal protein L24p (L26e) | 1.285 | | 0.002 | | |  | | |  | |
| FORC16_0216 | LSU ribosomal protein L5p (L11e) | 1.704 | | 6.860 E-05 | | |  | | |  | |
| FORC16_0217 | SSU ribosomal protein S14p (S29e) / SSU ribosomal protein S14p (S29e), zinc-independent | 2.136 | | 2.865 E-05 | | |  | | |  | |
| FORC16_0218 | SSU ribosomal protein S8p (S15Ae) | 2.393 | | 3.853 E-04 | | |  | | |  | |
| FORC16_0219 | LSU ribosomal protein L6p (L9e) | 2.721 | | 2.788 E-04 | | |  | | |  | |
| FORC16_0220 | LSU ribosomal protein L18p (L5e) | 3.012 | | 1.407 E-04 | | |  | | |  | |
| FORC16_0221 | SSU ribosomal protein S5p (S2e) | 3.185 | | 0.001 | | |  | | |  | |
| FORC16_0222 | LSU ribosomal protein L30p (L7e) | 3.25 | | 7.540 E-05 | | |  | | |  | |
| FORC16_0223 | LSU ribosomal protein L15p (L27Ae) | 3.026 | | 0.001 | | |  | | |  | |
| FORC16_0225 | LSU ribosomal protein L36p | 2.647 | | 6.654 E-05 | | |  | | |  | |
| FORC16_0226 | SSU ribosomal protein S13p (S18e) | 1.319 | | 0.0003 | | |  | | |  | |
| FORC16_0227 | SSU ribosomal protein S11p (S14e) | 1.745 | | 0.001 | | |  | | |  | |
| FORC16_0228 | SSU ribosomal protein S4p (S9e) | 2.217 | | 7.322 E-07 | | |  | | |  | |
| FORC16_0230 | LSU ribosomal protein L17p | 3.105 | | 0.001 | | |  | | |  | |
| **Nucleotide metabolism** | | | | | | | | | | | |
| FORC16_0413 | Carbamoyl-phosphate synthase small chain | 1.11 | | 0.007 | | |  | | |  | |
| FORC16_0414 | Carbamoyl-phosphate synthase large chain | 2.287 | | 0.002 | | |  | | |  | |
| FORC16_3108 | N-Ribosylnicotinamide phosphorylase | 3.582 | | 0.00047421 | | | 1.471 | | | 0.015 | |
| FORC16_0682 | Ribose-phosphate pyrophosphokinase | 1.58 | | 0.02 | | |  | | |  | |
| FORC16_0691 | UDP-sugar hydrolase; 5'-nucleotidase | 2.773 | | 0.001 | | |  | | |  | |
| **Cell cycle** | | | | | | | | | | | |
| FORC16_0397 | Cell division protein FtsI | 1.491 | | 0.027 | | | 1.497 | | | 0.03 | |
| FORC16_0803 | Cell division topological specificity factor MinE | 1.186 | | 0.027 | | | 1.517 | | | 0.046 | |
| FORC16_0852 | Cell division trigger factor | 2.205 | | 0.005 | | | 2.714 | | | 0.03 | |
| FORC16_0741 | Cell division inhibitor Slr1223 | -1.327 | | 0.005 | | |  | | |  | |
| **Adherence** | | | | | | | | | | | |
| FORC16_2527 | MSHA biogenesis protein MshK | -0.872 | | 0.011 | | |  | | |  | |
| FORC16_2528 | MSHA biogenesis protein MshJ | -0.937 | | 0.005 | | |  | | |  | |
| FORC16_2529 | MSHA biogenesis protein MshI | -1.008 | | 0.002 | | |  | | |  | |
| FORC16_2530 | MSHA biogenesis protein MshH | -1.14 | | 0.002 | | |  | | |  | |
| FORC16_2372 | Type IV pilin | -1.23 | | 0.011 | | | -1.371 | | | 0.029 | |
| FORC16_2373 | Type IV fimbrial assembly, ATPase PilB | -1.277 | | 0.008 | | | -1.139 | | | 0.014 | |
| FORC16_2374 | Type IV fimbrial assembly protein PilC |  | |  | | | -0.913 | | | 0.038 | |
| FORC16_3424 | Chitin binding protein | 0.925 | | 0.005 | | |  | | |  | |
| **Iron uptake** | | | | | | | | | | | |
| FORC16_4281 | Vibriolysin, extracellular zinc protease | -1.225 | | 0.003 | | |  | | |  | |
| FORC16_0126 | Ferric siderophore transport system, biopolymer transport protein ExbB |  | |  | | | -2.721 | | | 0.043 | |
| FORC16_4135 | Catechol siderophore ABC transporter, substrate-binding protein |  | |  | | | -0.885 | | | 0.041 | |
| FORC16_4127 | 2,3-dihydro-2,3-dihydroxybenzoate dehydrogenase | -1.067 | | 0.002 | | |  | | |  | |
| FORC16_4128 | Isochorismate synthase of siderophore biosynthesis | -1.316 | | 0.028 | | |  | | |  | |
| FORC16_4129 | 2,3-dihydroxybenzoate-AMP ligase | -1.583 | | 0.016 | | | -1.165 | | | 0.032 | |
| FORC16_4133 | 2,3-dihydroxybenzoate-AMP ligase | -1.477 | | 0.012 | | | -1.691 | | | 0.011 | |
| FORC16_4134 | Aryl carrier domain | -1.319 | | 0.011 | | |  | | |  | |
| FORC16_0789 | Ferrous iron transport protein B | -2.132 | | 0.003 | | |  | | |  | |
| FORC16_0790 | Ferrous iron transport protein C | -2.097 | | 0.01 | | |  | | |  | |
| FORC16_1743 | Non-specific DNA-binding protein Dps / Iron-binding ferritin-like antioxidant protein / Ferroxidase | -2.107 | | 0.008 | | |  | | |  | |
| FORC16_3373 | Ferric iron ABC transporter, ATP-binding protein | -1.275 | | 0.014 | | | -1.856 | | | 0.005 | |
| FORC16_3374 | Ferric iron ABC transporter, permease protein | -1.239 | | 0.032 | | | -1.16 | | | 0.047 | |
| FORC16_3953 | Heme O synthase, protoheme IX farnesyltransferase COX10-CtaB | -3.803 | | 0.004 | | | -2.401 | | | 0.003 | |
| FORC16_3954 | Heme A synthase, cytochrome oxidase biogenesis protein Cox15-CtaA | -3.974 | | 0.009 | | | -2.797 | | | 0.003 | |
| FORC16_3705 | TonB-dependent heme and hemoglobin receptor HutA ; TonB-dependent hemin , ferrichrome receptor | -1.47 | | 0.001 | | |  | | |  | |
| FORC16_3491 | Iron ABC transporter | -0.927 | | 0.041 | | |  | | |  | |
| FORC16_3492 | Ferric vibriobactin, enterobactin transport system, permease protein VctG | -0.936 | | 0.019 | | |  | | |  | |
| FORC16_3493 | Ferric vibriobactin, enterobactin transport system, ATP-binding protein | -1.099 | | 0.047 | | |  | | |  | |
| **RTX toxin** | | | | | | | | | | | |
| FORC16_3881 | RTX toxins determinant A and related Ca2+-binding proteins | -0.981 | | 0.046 | | | -1.557 | | | 0.013 | |
| FORC16_3884 | RTX toxin transporter | -0.868 | | 0.004 | | |  | | |  | |

**Supplementary Figure S1.** Unique genomic regions of *V. vulnificus* FORC_016. The unique genomic region of *V. vulnificus* FORC_016 was compared with a similar region of *V. vulnificus* MO6-24/O. The arrows represent the coding regions of (A) CPS gene cluster, (B) enterobactin receptor, and (C) heme receptor in *V. vulnificus* FORC_016. The middle line indicates amino acid sequence homology of each gene pair. The unique genes in *V. vulnificus* FORC_016 was highlighted with orange color. The figure was derived using the nucleotide sequences of *V. vulnificus* FORC_016 (CP011775, CP011776), and *V. vulnificus* MO6-24/O (CP002469 and CP002470).


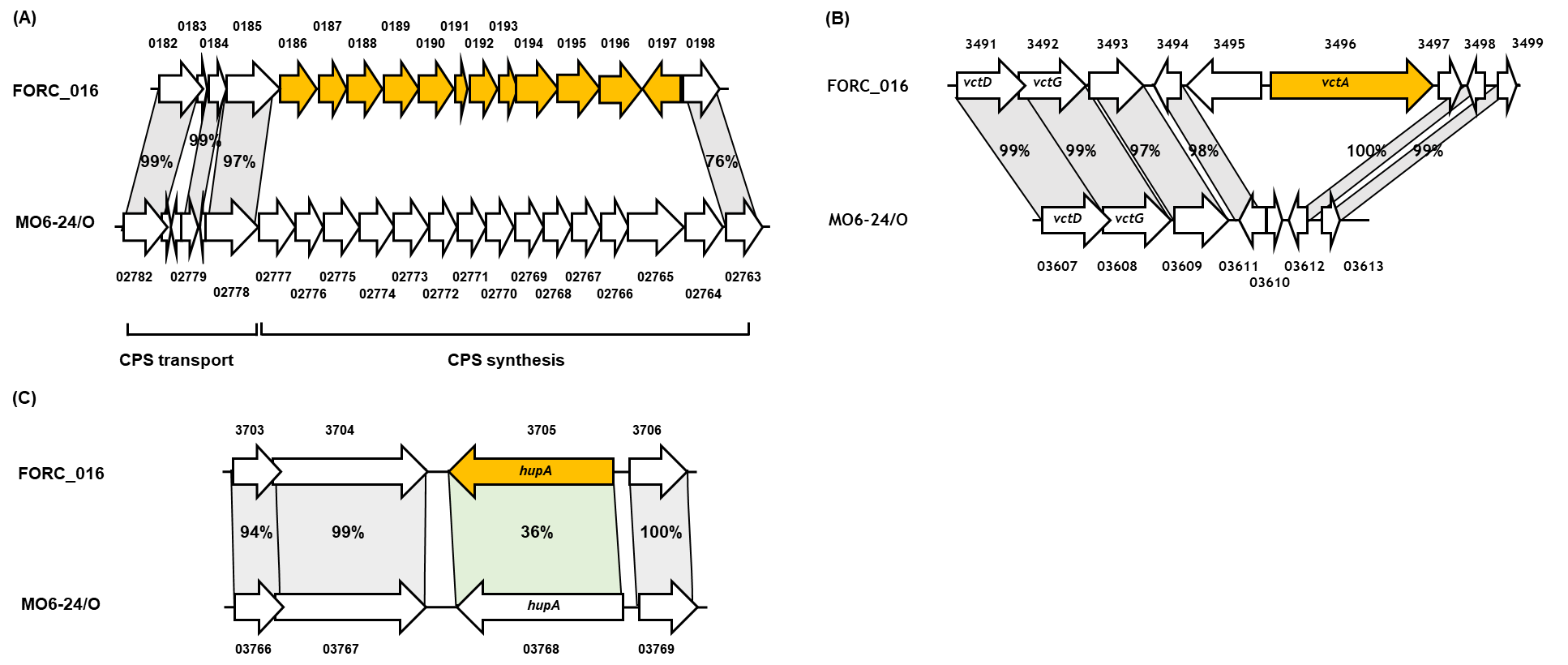

Supplement: Supplementary file 2 [file Data_Sheet_1.docx]
